# Coupled mesoporous silica nanoparticles and limonene–chitosan Pickering emulsions: enhanced insecticidal delivery and selectivity

**DOI:** 10.1186/s12951-026-04172-0

**Published:** 2026-03-19

**Authors:** Nannan Lv, Zhixuan Xu, Qingyang Zhang, Xinyu Wang, Yunxuan Wu, Ren Li, Jianting Fan

**Affiliations:** 1 School of Forestry and Biotechnology, National Joint Local Engineering Laboratory for High-Efficient Preparation of Biopesticide, Zhejiang A & F University, Lin’an, 311300 China; 2 Institute of Plant Protection, Beijing Academy of Agriculture and Forestry Sciences, Beijing, 100097 China

**Keywords:** Nanodelivery, Porous nanocarriers, Pickering emulsions, Bioactive adjuvants, Targeting

## Abstract

**Background:**

Nanodelivery represents a promising strategy to enhance pesticide efficacy while reducing associated environmental risks. Mesoporous silica nanospheres (MSNs) can serve as effective nanocarriers but improvements are essential in terms of targeting and efficiency. A promising approach involves combining nanocarriers-mediated pesticide delivery system with bioactive compounds, in which the compounds act as synergist that can disrupt insect defence mechanisms. Pickering emulsions (PEs) offer significant advantages in improving the bioavailability of active ingredients from compounds, which would enhance the synergistic effect to insecticides. Beyond encapsulating bioactive compounds, PEs serve as efficient pesticide delivery vehicles, enhancing leaf wettability and foliar deposition, making them effective adjuvants. This study aimed to construct a hybrid platform that integrates pesticides-loaded MSNs with synergistic PE as adjuvants, developing porous nanocarrier-based nanoemulsions to fight against insecticide-resistant pests.

**Results:**

The nanosystem (IMI@MSNs@lim@chs) that integrates imidacloprid (IMI)-loaded MSNs with limonene–chitosan (lim@chs) Pickering emulsions as adjuvants was developed and successfully control the resistance agroforestry pests, *Myzus persicae* (Sulzer). The IMI@MSNs complex showed increased toxicity than IMI, which account for the pesticide absorption and delivery capacity of MSNs. While incorporation IMI@MSNs with lim@chs, the IMI@MSNs@lim@chs nanosystem achieved the highest efficacy, indicating the remarkable synergistic effect of the lim@chs adjuvant. Mechanistic analyses have demonstrated that the lim@chs enhanced IMI activity was attributed to remarkable antibacterial effects, hydrophobicity, and lipophilicity, which suppress P450-mediated detoxification by inducing gut microbiota deficiency and facilitating epidermal penetration *via* cuticle disruption. In parallel, the porous MSNs promote nanosystem adhesion, preventing run-off and improving the IMI recovery rate after leaching. The lipophilic lim@chs enhances leaf wettability *via* supramolecular interactions, benefiting droplet rebound and promoting foliar deposition. Importantly, IMI@MSNs@lim@chs exhibits reduced toxicity and minimal impact on the predation capacity of *Coccinella septempunctat*, demonstrating the lower environmental risks.

**Conclusions:**

The findings of this study demonstrate an effective synergistic coupling of porous nanocarriers with Pickering emulsions as adjuvants to enhance pesticide delivery and selectivity, offering a promising platform for sustainable pest management.

**Graphical abstract:**

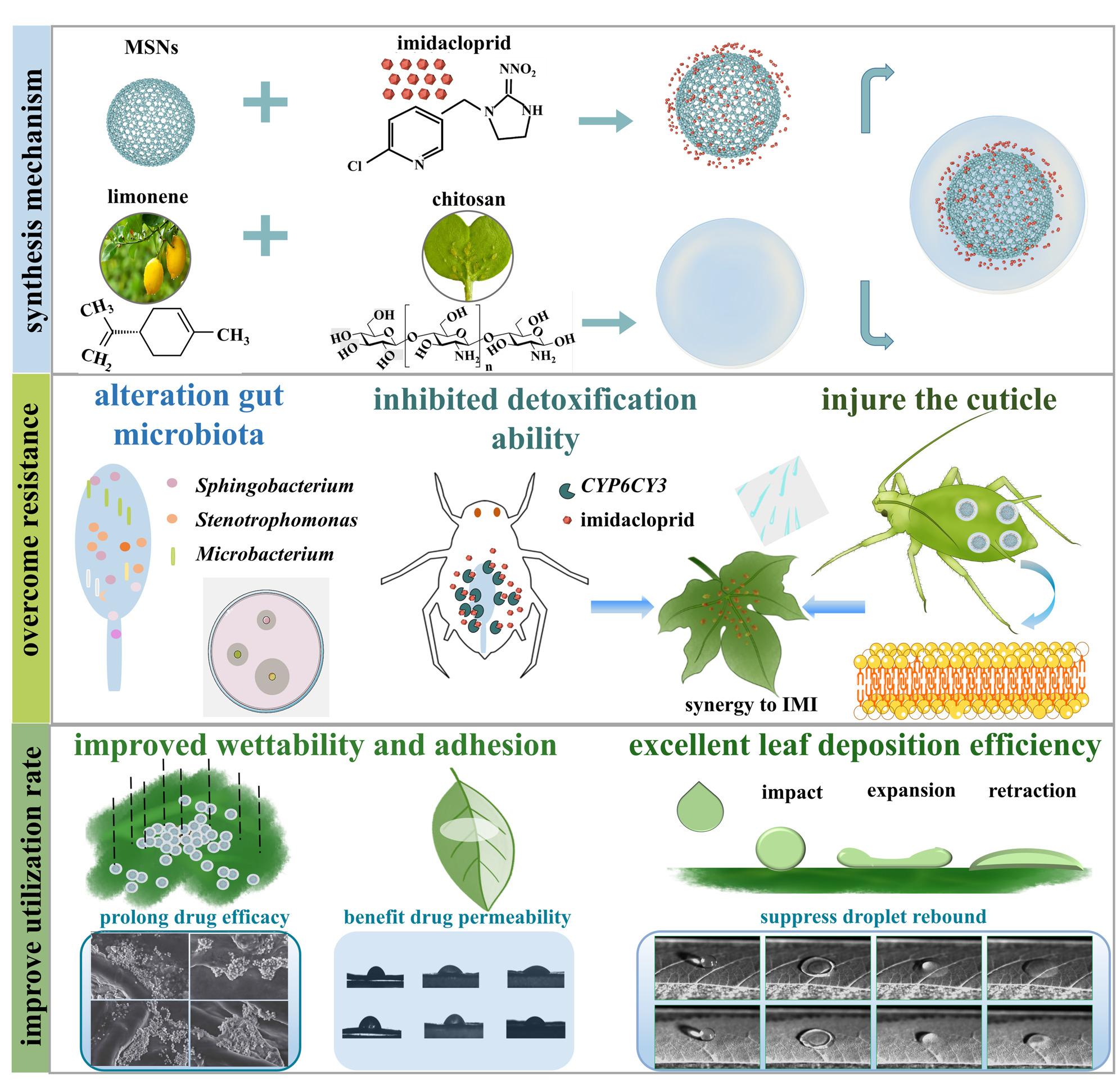

**Supplementary Information:**

The online version contains supplementary material available at 10.1186/s12951-026-04172-0.

## Introduction

Nanocarrier-mediated pesticide delivery is emerging as an effective strategy to enhance pesticide efficacy while minimizing environmental risks. Mesoporous silica nanospheres (MSNs), exhibiting highly ordered pores, large surface areas, and tunable release properties, have been widely studied as potential pesticide carriers [1]. MSN-based delivery systems improve pesticide utilization by facilitating controlled release, prolonging foliar retention, and reducing photodegradation [2–4]. Previous examples, such as ace@MSNs and avm@MSNs, significantly enhanced the insecticidal activity of acetamiprid (ace) and avermectin (avm) compared to their conventional application [5, 6]. However, there is a pressing need to improve the targeting specificity and field-level efficiency of the MSNs-mediated delivery systems.

The combination of nanocarriers with bioactive compounds that can disrupt insect defense mechanisms represents a promising approach. The antibacterial active ingredients exhibit a synergistic effect with insecticides due to the fact that the deficiency of gut microorganisms is associated with the decreased detoxification ability of insects [7]. Insects rely heavily on gut symbiotic microorganisms to regulate detoxification pathways. Disruption of these symbionts has been shown to suppress cytochrome P450 activity, thereby increasing insect susceptibility to xenobiotics [8]. The mechanism involves the modulation of P450 enzyme activity by microbial-generated reactive oxygen species (ROS), where a reduced abundance of gut microorganisms lowers ROS levels, suppressing detoxification metabolism and increasing insecticide vulnerability [9]. Thus, the design of microbiota-targeting synergist to increase pesticide bioavailability was promising in addressing the insect pests. Limonene (lim), a volatile monoterpene abundant in citrus peels, exhibits a broad-spectrum antimicrobial activity and lower environmental toxicity [10]. Moreover, it significantly enhanced the insecticidal activity of pesticides against aphids, whiteflies, and fruit flies, delivering significant field efficacy [11, 12]. However, agricultural applications are hindered by instability in aqueous solutions, rapid degradation under light and heat, and poor membrane permeability.

Encapsulation technologies, particularly Pickering emulsions (PEs), offer several key advantages in enhancing oil-based systems, particularly in terms of stability, controlled release, bioavailability [13]. Chitosan, a natural biopolymer with antimicrobial properties, has been widely used to fabricate stable PEs [14]. Chitosan-stabilized limonene emulsions (lim@chs) enhance limonene stability, viscosity, and thermal resistance, improving overall bioavailability [15]. Here, the lim@chs Pickering emulsions-targeting the gut microbiota was proposed as adjuvants of the MSNs-mediated pesticide delivery systems to enhance the insecticides activity. In addition to encapsulating bioactive compounds, PEs can serve as excellent pesticide delivery vehicles with the positive leaf wettability and foliar deposition efficient, making them promising candidates as adjuvants [16]. Thus, the lim@chs Pickering emulsions as adjuvants have great potential synergistic effect when combined with MSNs-mediated pesticide delivery systems.

Hence, a hybrid nanosystem (IMI@MSNs@lim@chs) that integrates imidacloprid (IMI)-loaded MSNs with lim@chsPickering emulsions as adjuvants was designed. The IMI@MSNs complexes were expected in absorbing and delivering IMI, lim@chs was designed to suppress the detoxification capacity of insect pests and enhance MSNs-mediated IMI delivery efficiency. The proposed multifunctional platform was validated by treating the green peach aphid, *Myzus persicae* (Sulzer), a globally persistent destructive agroforestry pest. The *M. persicae* infests more than 500 host plant species, damaging crops through direct feeding and virus transmission, and has developed widespread resistance to neonicotinoids [17, 18]. Here, the nanosystem IMI@MSNs@lim@chs was employed to addresses resistance of *M. persicae*, through disrupt the resistance mechanism and improve pesticide targeting.

We have evaluated the insecticidal activity of IMI@MSNs and IMI@MSNs@lim@chs against *M. persicae*, analyzing the role of lim@chs in suppressing gut microbiota and P450-mediated detoxification, and assessing the effects on cuticle disruption and penetration efficiency. In addition, we have assessed foliar adhesion, wettability, and deposition efficiency to establish field application potential. In terms of application safety, we have measured toxicity and predation capacity in the case of *Coccinella septempunctata*. The findings of this work have established a cooperative nanodelivery platform that leverages porous nanocarriers and microbiota-targeting Pickering emulsions to enhance pesticide efficacy and selectivity, offering a novel sustainable strategy for pest management.

## Materials and methods

### Materials and insects

Geographical populations of *Myzus persicae* were collected from three different areas of China in 2024. The sample locations were Lincang City of Yunnan Province (YNLC), Zhengzhou City of Henan Province (HNZZ), and Jimo City of Shandong Province (SDJM), respectively. The aphids were reared on radish seedlings, *Raphanus sativus*, in controlled conditions of 20–23^◦^C, 60–70% relative humidity, and a photoperiod of 16:8 h (light: dark). Mesoporous nano silica (MSNs) was purchased from Beike Nanotechnology Co., Ltd. (SuZhou, China). Chitosan, limonene, Tween 80 and glycerol were obtained from Macklin Agriculture Chemical Ltd. (Shanghai, China). Imidacloprid (technical grade 95.3%) was obtained from DuPont Agriculture Chemical Ltd. (Shanghai, China).

### Preparation and characterization IMI@MSNs@lim@chs

A sample (0.5 mg) of MSN was dissolved in 5 mL phosphate-buffered saline (PBS, pH 7.4), followed by the addition of 0.5 mg imidacloprid. The mixture was sonicated (power: 300 W, temperature: 25 ± 2 °C) for 10 min and then shaken at 200 rpm for 4 h. The IMI@MSNs was recovered by centrifugation at 10,000 rpm for 5 min, washed with deionized water, and vacuum-dried to obtain the IMI@MSNs complexes.

In the preparation of limonene-chitosan (lim@chs) Pickering emulsions, 5 g chitosan was dissolved in 50 mL 1% (v/v) aqueous acetic acid and allowed to stand overnight at room temperature to ensure complete dissolution. Then, 800 mL distilled water was added with 2 mL Tween 80 as surfactant and 5 mL glycerol as plasticizer. The mixture was stirred at 45 °C for 2 h to achieve a homogeneous solution. Limonene was then added dropwise to the continuously stirred chitosan mixture at 13,000 rpm, forming a stable oil-in-water (O/W) emulsion. The final volume of the lim@chs system was adjusted to 1000 mL with distilled water. In the preparation of the IMI@MSNs@lim@chs nanosystem, IMI@MSNs were incorporated in the lim@chs Pickering emulsion containing 20% (v/v) limonene, followed by sonication for 10 min and further stirring at 13,000 rpm for 30 min to ensure uniform dispersion.

The morphology of lim@chs was assessed by optical microscopy (Carl Zeiss Axio lmager A2, Germany). Scanning electron microscopy (SEM, ZEISS GeminiSEM 300, Germany) was conducted at an accelerating voltage of 5 kV after sputter-coating the samples with gold, to determine the morphology of MSNs, IMI@MSNs and IMI@MSNs@lim@chs. Transmission electron microscopy (TEM, JEOL JEM-F200, Japan) analyses were performed at 200 kV to examine the microstructure of samples. Elemental composition was analysed by TEM-coupled energy-dispersive X-ray spectroscopy (EDS) using a JED-2300 T instrument (JEOL, Japan) under the same TEM operating conditions. The zeta potential was determined using a Malvern Zetasizer (Malvern Instruments Ltd., UK); each sample was measured in triplicate at 25 °C. Fourier Transform Infrared (FTIR) spectroscopy was performed using a Thermo Fisher Scientific (USA) spectrometer. The data was recorded in the 4000 to 400 cm⁻¹ range with 64 scans and a resolution of 2 cm⁻¹.

### Physical stability assessment of IMI@MSNs@lim@chs

The particle size of lim@chs and IMI@MSNs@lim@chs for different storage periods and temperatures was measured in triplicate at 25 °C using a Malvern Zetasizer (Malvern Instruments Ltd., UK). The encapsulation efficiency (EE) of limonene in the IMI@MSNs@lim@chs nanosystem and lim@chs Pickering emulsions was also measured. In the lim@chs Pickering emulsions, the free limonene was collected and sampled for gas chromatography (GC) analysis using a flame ionization detector (FID). All the experiments were conducted in triplicate to ensure reproducibility. The GC was calibrated using a series of standard solutions of pure limonene to generate calibration curves (peak area vs. concentration). The concentration of limonene in each sample was determined from the GC peak area using the corresponding calibration curve. The limonene loading was obtained from the initial slope of the peak area-concentration curve: EE (limonene)= −Δn(limonene)/*n* × 100%, where − Δn(limonene) represents reactant consumption and “n” denotes the initial concentration of limonene.

### IMI loading and release measurements

High-performance liquid chromatography (HPLC) was conducted to quantify the imidacloprid loading on MSNs. A 5.0 mg sample of MSNs powder was dispersed in imidacloprid solutions at concentrations of 62.5, 125.0, 250.0, 500.0, and 1000.0 mg/L to prepare IMI@MSNs. The imidacloprid in the supernatant was collected for HPLC analysis (Agilent 1260 series) at a wavelength of 269.0 nm using a Zorbax SB-Aq C18 column (5.0 μm, 4.6 mm ×250.0 mm; Agilent). The mobile phase consisted of 75.0% ultrapure water and 25.0% acetonitrile, with a flow rate of 1.0 mL/min. A standard curve for imidacloprid was established to determine the concentration. The adsorption capacity of MSNs for imidacloprid was evaluated following the method described by Lv et al. (2024) [19]. For release measurement, an accurately weighed quantity of IMI@MSNs was dispersed in water and then shaken at 25 °C. At different time intervals, 1 ml samples were removed and centrifuged to obtain a clear supernatant. The IMI in the supernatant was the released amount from the MSNs, which was analysed by HPLC according to the above method. The IMI loading and release measurements were performed in triplicate.

### Toxicity bioassays on *Myzus persicae*

The leaf-dipping method with slight modifications was employed to assess the toxicity of limonene, imidacloprid, IMI@MSNs and IMI@MSNs@lim@chs to *Myzus persicae*. The IMI@MSNs and IMI@MSNs@lim@chs were diluted to the required concentrations using distilled water containing 0.05% (v/v) Triton X-100 for the bioassays. Cabbage leaf discs (20 mm diameter) were immersed in serial concentrations of imidacloprid, IMI@MSNs, or IMI@MSNs@lim@chs for 15 s. A control treatment was conducted with 0.05% (v/v) Triton X-100 in water. After treatment, the leaf discs were placed on disposable PE gloves to air dry and then inverted onto agar beds in 12-well cell culture plates. Adult *M. persicae* aphids were carefully transferred onto the treated discs and covered with Chinese art paper to prevent escape. The bioassays were conducted at 21–23 °C with a 16:8 h light: dark photoperiod. Each concentration was tested with three replicates, and at least 30 aphids per replicate. Mortality was assessed 48 h post-treatment. The LC_50_ values were calculated using probit analysis with POLO Plus 2.0 statistical software (LeOra Software Inc., Berkeley, CA).

### 16 S rRNA analysis of the gut microbiota in *Myzus persicae* after treatment with lim@chs

Wingless adult aphids were carefully transferred onto cabbage leaf discs, followed by spraying with lim@chs Pickering emulsions containing 20% (v/v) limonene. As a control, wingless adult aphids were treated with distilled water. Following two days of exposure, the guts of the aphids were collected for 16 S rRNA sequencing. To remove surface bacteria, the aphids were first soaked in 75% ethanol for 90 s. Then, the entire gut was dissected under a stereomicroscope and transferred into centrifuge tubes containing DNA extraction buffer. For each replicate, 50 guts were dissected, and five replicates performed for each treatment. DNA extraction was carried out using a commercial DNA extraction kit (Qingke, Beijing, China), and the extracted DNA was subjected to high-throughput sequencing of the 16 S rRNA gene V4 region at Novogene (Beijing, China), following established protocols [20]. The primers, 505 F (5′- CCTAYGGGRBGCASCAG-3′) and 806R (5′-GGACTACNNGGGTATCTAA-3′), were used to amplify the V4 variable regions of the 16 S rRNA gene.

### Antibacterial activity of lim@chs against *Myzus persicae* gut bacteria

The gut symbiont was isolated and identified following the procedure reported by Lv et al. (2023) [21]. The antibacterial activity of lim@chs with respect to gut microbiota *Sphingomonas*, *Stenotrophomonas*, and *Microbacterium* of *M. persicae* was assessed using a Bacterial Staining Kit (SOLARBIO, Beijing, China). Bacterial suspensions were incubated with a staining solution (1 µL NucGreen, 2 µL EthD-III, and 8 µL NaCl) in the dark at room temperature for 15 min. Viable bacteria displayed green fluorescence, whereas dead bacteria exhibited red fluorescence, and were visualized using a laser confocal microscope (ZEISS LSM880, Germany).

Bacterial suspensions (1.5 × 10⁶ CFU/mL) were prepared to measure the minimum inhibitory concentration (MIC) of lim@chs against gut symbiotic bacteria. In a 96-well plate, 100 µL bacterial suspension was added to each well, followed by 100 µL lim@chs containing 5%, 10%, 15%, or 20% (v/v) limonene. The control groups were treated with 200 µL bacterial suspension without lim@chs, and 100 µL bacterial suspension mixed with 100 µL sterile water. The plates were incubated, and the bacterial concentrations of *Sphingomonas*, *Stenotrophomonas*, and *Microbacterium* were measured every 3 h to assess growth inhibition and determine the MIC of lim@chs. The MIC of tetracycline to *Sphingomonas*, *Stenotrophomonas*, and *Microbacterium* was also observed for a standard antibiotic reference control. The MIC of tetracycline and lim@chs to these three genera of gut bacteria was measured in triplicate.2.8 Transcriptomic analysis of *Myzus persicae* after treatment with lim@chs.

After treating apterous adult *M. persicae* with lim@chs (20% (v/v) limonene) for two days, the total RNA was extracted in triplicate using TRIzol^®^ reagent (Invitrogen, Carlsbad, CA, USA) according to the manufacturer’s protocol (20 aphids per tube). The RNA was then sequenced on an Illumina NovaSeq platform to obtain high-quality data. Clean reads were mapped to the reference genome using HISAT2 v2.0.5. Gene expression levels were quantified based on the fragments per kilobase of exon per million mapped reads (FPKM). Differentially expressed genes (DEGs) were identified using the DESeq2 R package, applying a false discovery rate (FDR) threshold < 0.05. Volcano plots were generated to visualize the distribution of DEGs, and KEGG enrichment analysis was conducted to identify the biological pathways associated with the DEGs.

### Re-introduction gut bacteria to lim@chs-treated *Myzus persicae*

In the gut bacteria re-introduction experiments, strains of *Sphingomonas*, *Stenotrophomonas*, and *Microbacterium* were first cultured in 2×YT liquid medium at 27 °C with shaking at 200 rpm until reaching an OD600 of 0.8. Subsequently, 100 apterous adult aphids from the YNLC population, previously treated with lim@chs, were gently transferred onto cotton leaf discs (80 mm in diameter). Each disc was then evenly sprayed with 50 µL of bacterial inoculum of these three genera of gut bacteria, respectively. Aphids in the control group were treated with an equivalent volume of sterilized water. All treatments were performed in triplicate. After five days of bacterial exposure, total RNA was extracted for analysis of P450 gene expression, and aphid susceptibility to imidacloprid was assessed using the leaf-dipping method.

### Quantitative RT-PCR of genes related to P450 and cuticle protein

 The total RNA was extracted from apterous adult aphids (20 aphids per tube), and first-strand cDNA was synthesized from 1.0 µg RNA using the PrimeScript RT reagent kit with gDNA Eraser (Takara Biotechnology, Dalian, China). Gene amplification was conducted using ChamQ SYBR qPCR Master Mix (Vazyme, Nanjing, China) on an Applied Biosystems^™^ 7500 Real-Time PCR System (Thermo Fisher, Massachusetts, USA) with the following cycling conditions: initial denaturation at 95 °C for 30 s, followed by 40 cycles at 95 °C for 10 s (denaturation) and 60 °C for 30 s (annealing/extension). A melt curve analysis was performed at 95 °C for 15 s, 60 °C for 10 s, and 95 °C for 15 s. Three biological replicates were generated, each with three technical replicates. The annotation of genes and primers used in this study are given in Table S1 and S2. The reference sequence of genes for this study was listed in the Supplementary information. Relative gene expression levels were quantified using the 2^(-ΔΔCt) method [22]. 

### The observation on the cuticle of *Myzus persicae*

Changes to the cuticle of apterous adult aphids (*M. persica*) following lim@chs (20% (v/v) limonene) treatment were determined by SEM. Each aphid was mounted on conductive carbon glue and sputter-coated with gold at a current of 10 mA for 60 s. The samples were transferred to the sample chamber of a Quanta 450FEI Environmental Scanning Electron Microscope (FEI, Hillsboro, USA) for cuticle surface visual analysis. Each treatment involved three independent replicates.

Paraffin sections for *M. persica* before and after treatment with lim@chs (20% (v/v) limonene) were prepared. Initially, the aphid was fixed in 4% paraformaldehyde for 24 h to preserve the cellular structures. Following fixation, the aphid underwent dehydration using a graded series of alcohol solutions, ranging from 70% to 100% ethanol, to remove water. The tissue was then treated with xylene to replace alcohol and facilitate paraffin infiltration. The resultant aphid tissue was embedded in molten paraffin at 60 °C. Following solidification, the paraffin block was sectioned into thin slices using a microtome. The paraffin sections were stained with hematoxylin and eosin (H&E) to highlight cellular structures, and mounted on slides using Pannoramic MIDI II (3DHISTECH Ltd, Hungary).

### Analysing the content of Imidacloprid in *Myzus persicae*

Apterous adult aphids from the YNLC populations were subjected to treatments with the LC_50_ of IMI@MSNs@lim@chs (10.0 mg/L), 10.0 mg/L IMI@MSNs, or 10.0 mg/L imidacloprid via spraying. For controls, *M. persicae* was treated with sterilized water. The aphids were sampled on the same day, 4 h after treatment; 100 mg of aphids from each replicate were then ground and extracted with 1000 µL of acetonitrile. Following this, the samples were subjected to rotary evaporation until reduced to 100 µL and subsequently purified using 0.45 μm filters for HPLC-MS analysis, according to the method in Lv et al. (2024) [19]. Each treatment was conducted in triplicate.

To determine the recovery efficiency of imidacloprid, equivalent masses of imidacloprid were added to either 1 mL of acetonitrile or 1 mL of the aphid homogenate diluted with acetonitrile. It is important to note that the 10 mg of aphids used in this part of the experiment had not been exposed to imidacloprid. The recovery efficiency of imidacloprid from the homogenate of aphids was calculated using the following equation: R=$\:\left(\frac{\mathrm{C}1}{\mathrm{C}2}\right)$×100%. The R is the recovery rate (%), C1 is the concentration of imidacloprid in the homogenization of cotton aphids, and C2 is the concentration of imidacloprid in acetonitrile.

### Adhesion, surface wettability and deposition efficiency measurement

The foliage retention of IMI@MSNs@lim@chs was evaluated in simulated rainwater washout tests. The IMI, IMI@MSNs, and IMI@MSNs@lim@chs suspensions were sprayed onto tender peach leaves and air-dried. Treated leaves were subjected to 1 mL deionized water sprayed at 0.2 mL/s; the control leaves were sprayed with deionized water. The recovery efficiency of imidacloprid was evaluated in triplicate using two parallel extraction protocols: an equal amount of imidacloprid was either dissolved directly in 1 mL of pure acetonitrile or applied to tender peach leaves followed by elution with 1 mL of acetonitrile. The washing process was repeated 0, 2, and 4 times, and the distribution of samples on the leaves was observed by SEM (ZEISS Sigma 360, Germany). Residual IMI was collected after each wash and recovered with acetonitrile. The remaining IMI was quantified by HPLC to assess the adhesion properties. Each treatment was repeated nine times.

The contact angles of 20 mg/L IMI, IMI@MSNs, and IMI@MSNs@lim@chs on the peach leaves were measured using an OCA20 unit (Data-Physics, Germany). Each sample was measured in five times. In addition, the droplet impact of IMI@MSNs@lim@chs was evaluated by releasing a 20 µL drop from a height of 55 cm onto the peach leaves. The impact event was recorded using a high-speed camera (Phantom Miro LC310, Vision Research Inc., USA) at 3631 fps with a 90° or 60° viewing angle. The recorded videos were analysed to track the evolution of droplet area.

### Insecticidal activity assessment in greenhouse

The insecticidal activity of the IMI@MSNs@lim@chs nanosystems against *M. persicae* was evaluated on radish seedling stage using the leaf-spraying method. The assessments were conducted under controlled greenhouse conditions, maintaining a temperature of 20–23 °C and a relative humidity of 60–70%. The measured LC_50_ of IMI for the HNZZ population was 11.3 mg/L, and this dose was used to assess the insecticidal efficacy of IMI and IMI@MSNs@lim@chs nanosystems. The IMI suspensions and IMI@MSNs@lim@chs nanosystems were uniformly sprayed onto the leaf surfaces of radish seedlings infested with aphids using a plastic spray tower. A control treatment consisting of water with 0.1% Triton-100 was applied to the plants. Each treatment was independently repeated six times, with at least thirty *M. persicae* per replicate. The mortality of *M. persicae* in each treatment was recorded daily.

### Toxicity bioassays on *Coccinella septempunctata*

A pesticide-impregnated filter paper method was employed to access the insecticidal activity of IMI@MSNs@lim@chs against *C. septempunctata* larvae [23]. The concentrations of IMI and IMI@MSNs@lim@chs were adjusted through serial dilution with distilled water containing 0.05% (v/v) Triton X-100 and lim@chs (20% (v/v) limonene) for bioassay testing. Filter papers were placed at the bottom of Petri dishes (9 cm diameter and 2 cm height) and treated with 1 mL IMI or IMI@AgNps@CMSNs. Subsequently, third-instar *C. septempunctata* larvae were transferred onto the treated filter papers and provided with sufficient aphid food. Mortality rates were recorded 24 and 48 h post-treatment. Each concentration was tested with 10 larvae, and the experiment was repeated three times. The LC_50_ values were calculated based on the method outlined in the section of toxicity bioassays on *Myzus persicae*.

### Evaluation of predatory behaviour on *Coccinella septempunctata*

In this study, the LC_10_ and LC_50_ values of IMI to *C. septempunctata* were 3 mg/L and 50 mg/L, respectively. These two sub-lethal doses of IMI or IMI@MSNs@lim@chs were used to treat *C. septempunctata* for 24 h using the pesticide-impregnated filter paper method. After treatment, *C. septempunctata* was transferred into a Petri dish (9 cm diameter and 2 cm height) with varying *M. persicae* densities of 25, 50, 100, 200, and 300 per dish. Each density treatment was repeated with five replicates; *C. septempunctata* treated with distilled water served as the control group. The predatory capacity of the ladybirds was calculated using the Holling method: [24]

$$\mathrm{N}_\mathrm{a}=\mathrm{aN}_0/\mathrm{(1+aT}_\mathrm{h}\mathrm{N}_0)$$

where N_a_ (head) represents the predacious number, N_0_ (head) denotes the prey density, a is the instantaneous rate of discovery, and T_h_ represents the test duration.

### Statistical analysis

 Statistical analysis was conducted using SPSS 21.0 (SPSS, Chicago, IL, USA). Prior to one-way ANOVA, normality (Shapiro-Wilk test) and homogeneity of variance (Levene’s test) were verified for all datasets (Table S3). Following significant ANOVA results, Tukey’s HSD post hoc test was applied for pairwise comparisons. For analyses involving multiple comparisons across populations, *p*-values were adjusted using the Benjamini-Hochberg false discovery rate (FDR) correction to control for Type I error (Table S4). Data visualization was performed with GraphPad Prism version 9.0 (GraphPad Software, San Diego, CA, USA).

## Results and discussion

### Characterization and physical stability assessment of lim@chs and IMI@MSNs@lim@chs

**Fig. 1:**
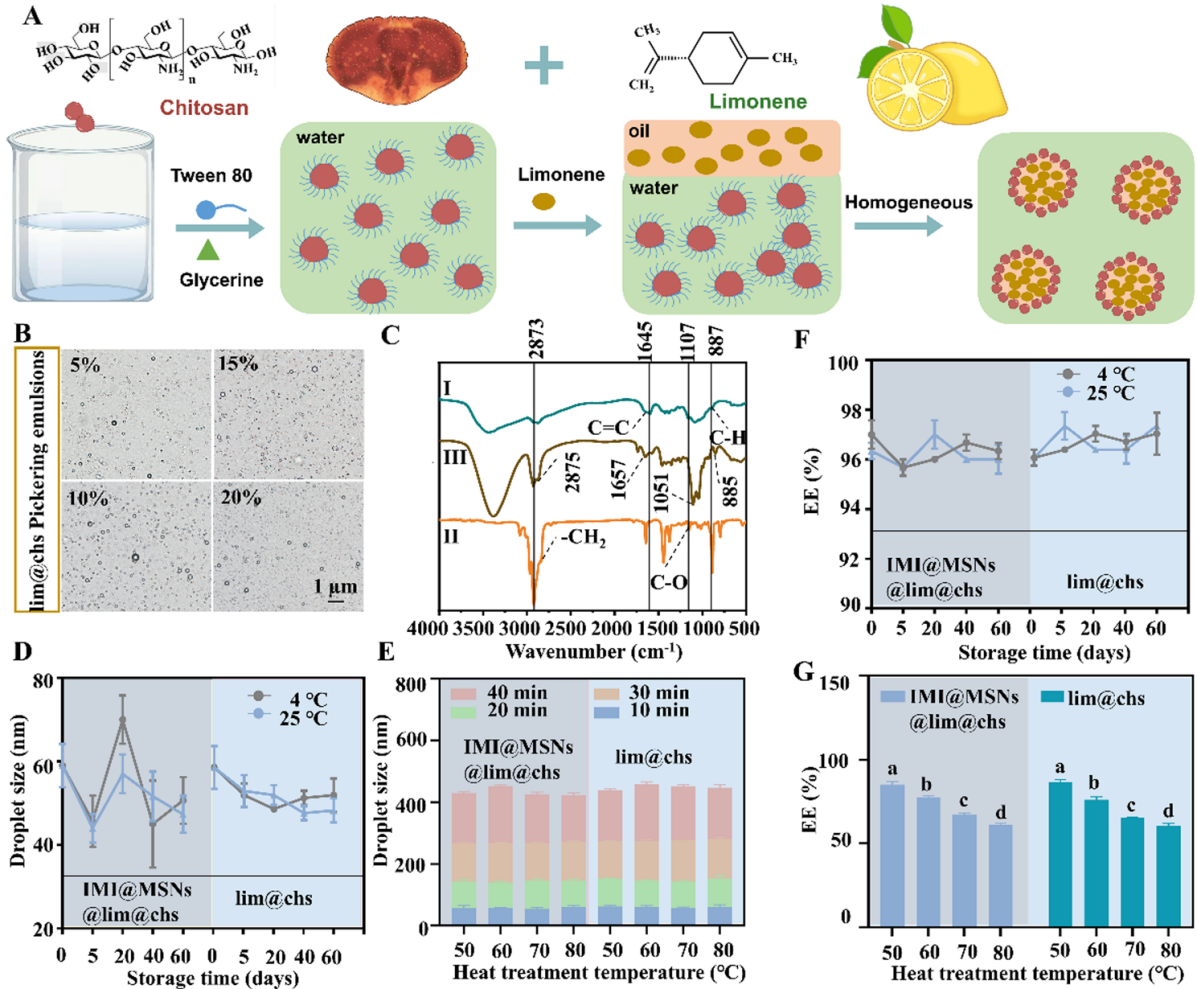
Synthesis and characterization of lim@chs Pickering emulsions, and physical stability assessment of lim@chs and IMI@MSNs@lim@chs. (**A**) Schematic representation of lim@chs Pickering emulsion preparation procedure. (**B**) Optical microscopy images of lim@chs with different concentrations of limonene. (**C**) FTIR spectra of (I) limonene, (II) chitosan, and (III) lim@chs. (**D**)-(**G**) The droplet size and EE (%) of lim@chs and IMI@MSNs@lim@chs. The bars with different lower-case letters are statistically significantly different applying a one-way ANOVA test, followed by a Tukey multiple comparison test (*P* < 0.05)

In this study, lim@chs were designed as adjuvants to enhance the insecticidal toxicity of imidacloprid to *M. persicae* (Fig. S1). As shown in Fig. 1A, the lim@chs were prepared by encapsulating limonene with chitosan. The morphologies of lim@chs containing 5%, 10%, 15% and 20% (v/v) limonene were characterized by optical microscopy (Fig. 1B). The Fourier-transform infrared (FTIR) spectra of limonene, chitosan and lim@chs have confirmed the characteristic chemical composition of the components and successful encapsulation (Fig. 1C). In the case of chitosan, the peaks at 1150 cm^− 1^ correspond to the C-O-C asymmetric stretching vibration. The bands 2825 cm^− 1^ was assigned to -CH_2_ stretching vibration. Limonene exhibits an absorption band at 1604 cm^− 1^ (C = C stretching vibration), and C-H bending vibration at 887 cm^− 1^. The FTIR spectrum of lim@chs confirms the presence of the chitosan matrix, as evidenced by its characteristic -CH_2_ stretching band at 2922 cm⁻¹ and C-O-C vibrations at 1107 cm⁻¹. Crucially, the retention of the signature limonene signals at 1649 and 851 cm⁻¹ demonstrates the successful encapsulation of limonene by chitosan. We have also quantified the FTIR peak intensities by OMNIC 9.3. The results indicated that the peak intensities for -CH₂, C = C, C-O-C, and C-H in lim@chs were 0.357, 0.140, 0.151, and 0.06, respectively. In comparison, the corresponding -CH₂ and C-O-C peak intensities in pure chitosan were 0.072 and 0.072, while the C = C and C-H peak intensities in pure limonene was 0.061 and 0.128. For chitosan FTIR spectrum, the -CH₂ and C-O peak intensities show 1:1 ratio. In the composite, this ratio shifts to 2:1 in lim@chs FTIR spectrum. Similarly, for limonene FTIR spectrum the C = C and C-H peak intensities ratio was 1:2, and it shifts to 2:1 in lim@chs FTIR spectrum. These significant changes in specific intensity ratios provide quantitative evidence for molecular interaction and successful encapsulation.

The IMI@MSNs complexes were prepared by loading imidacloprid on mesoporous silica nanospheres (MSNs) to improve delivery efficiency. The IMI@MSNs@lim@chs nanosystem was obtained by dispersing IMI@MSNs on lim@chs. The droplet size and encapsulation efficiency (EE) of IMI@MSNs@lim@chs and lim@chs (20% limonene) were monitored during 60 days of storage at 4℃ and 25℃ (Fig. 1D and F). Both systems exhibited good stability under these conditions, maintaining volume-average droplet sizes of 34.2–76.0 μm and high EE values (95–99%). The stability of lim@chs emulsions can be attributed to the emulsifying properties of chitosan, which facilitate interaction with both aqueous and oil phases, and the lipophilic nature of limonene that promotes droplet formation and stabilization [25, 26]. Furthermore, Optical microscopy revealed no significant coalescence or morphological change in droplet structure compared to the fresh lim@chs Pickering emulsions, over 20 days of quiescent storage at 25 °C (Fig. S2). In addition, the excellent physical stability observed during storage was consistent with the maintained Zeta potential values (8–11 mV), which suggested a stable interfacial structure (Fig. S3). These factors ensure long-term stability of the MI@MSNs@lim@chs nanosystem and lim@chs Pickering emulsions.

The effect of high temperatures on stability was also investigated. Following exposure to 50℃, 60℃, 70℃, and 80℃ for 10 min, both emulsions remained stable with no significant increase in droplet size. Prolonged heating resulted in a gradual droplet swelling, but both emulsions remained below 450 nm after 40 min at 80 °C (Fig. 1E). Elevated temperatures can reduce the oil and water interface and lower emulsifying efficiency [27]. In addition, chitosan swelling and the partial volatilization of limonene may contribute to droplet enlargement. Nevertheless, the chitosan thermal stability and the associated electrostatic barrier preserved the nanoemulsion structure [28]. The encapsulation efficiency decreased under high temperature treatment, but remained above 60% after 40 min at 80 °C (Fig. 1G). The observed reduction can be attributed to limonene volatility and droplet enlargement, which lower the oil-water interfacial area available for encapsulation [29, 30]. A high temperature can slightly reduce EE and increase droplet size, but the stabilizing effect of chitosan maintained the nanoemulsion state.

### Characterization of IMI@MSNs and IMI@MSNs@lim@chs

**Fig. 2:**
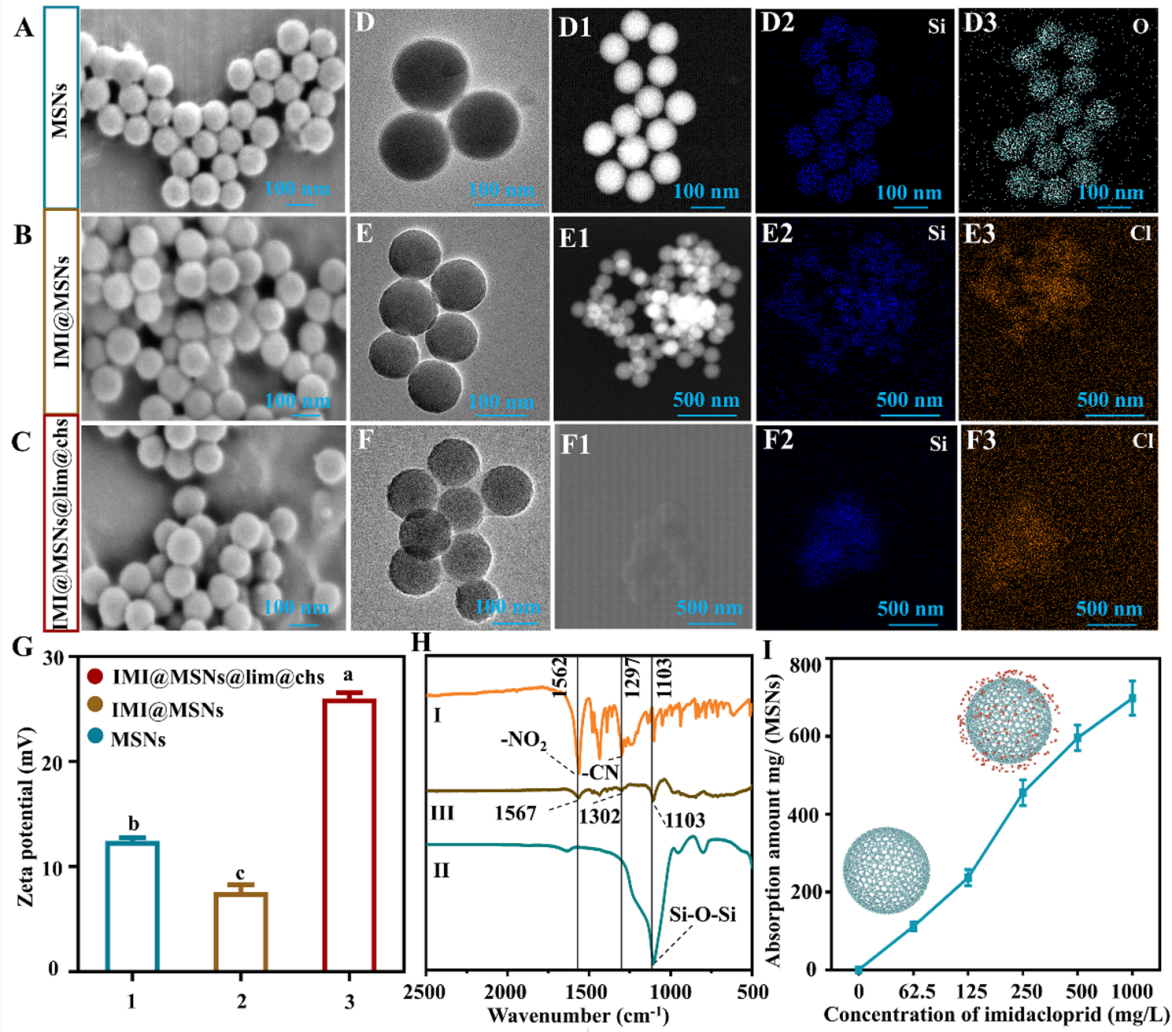
Characterization of the IMI@MSNs@lim@chs nanosystem. (**A**)-(**F**) SEM and TEM images, (D1) -(F3) Dark-field scanning TEM images with EDS mapping, (**G**) Zeta potential data for MSNs, IMI@MSNs and IMI@MSNs@lim@chs. The bars with different lower-case letters are statistically significantly different applying a one-way ANOVA test, followed by a Tukey multiple comparison test (*P* < 0.05). (**H**) FTIR spectra of (I) MSNs, (II) imidacloprid, and (III) IMI@MSNs. (**I**) Imidacloprid adsorption capacity of IMI@MSNs

The morphologies of MSNs, IMI@MSNs and IMI@MSNs@lim@chs were characterized by SEM (Fig. 2A and C) and TEM (Fig. 2D and F). The TEM-EDS elemental mapping has established that the silicon and chlorine content associated with MSNs (Fig. 2D2-2D3) and IMI are uniformly distributed on the surface of IMI@MSNs (Fig. 2E2-2E3) and IMI@MSNs@lim@chs (Fig. 2F2-2F3), confirming successful fabrication. The zeta potential measurements have shown that the value for MSNs decreased from 12.5 mV to 7.6 mV after loading IMI. It suggested an interaction between the pesticide and the carrier. The IMI@MSNs@lim@chs showed a significantly increased zeta potential (26.0 mV), indicating that the successful incorporation of lim@chs may preventing agglomeration and promoting uniform dispersal in solution (Fig. 2G). The FTIR spectra of MSNs, IMI and IMI@MSNs were also recorded to determine the characteristic surface chemical composition (Fig. 2H). The MSNs spectrum (II) exhibits a peak at 1103 cm^− 1^ that is attributed to Si-O-Si antisymmetric stretching. The presence of IMI in IMI@MSNs was confirmed by comparing the FTIR spectra of pure IMI and MSNs. In the case of the pure IMI spectrum (I), the -NO_2_ stretching vibration is detected at 1562 cm^− 1^, and the peaks for C-N stretching vibration appear at 1433 cm^− 1^, respectively. These characteristic peaks for IMI are consistent with previous reports [31, 32]. In the FTIR spectrum of IMI@MSNs, the retention of these key IMI signals at 1567 cm⁻¹ and 1435 cm⁻¹, without the emergence of new chemical bond formations, confirms the successful loading of IMI onto the MSN_S_. The adsorption capacity of MSNs for IMI was evaluated according to the stand curve of IMI (Fig. S4). The maximum IMI uptake by MSN is 698 mg/g at an IMI concentration of 1000 mg/L (Fig. 2I). By comparison, Nasif et al. (2025) reported that MCM-41, characterized by a uniform porous structure, exhibited a high adsorption capacity of 104.31 mg/g for IMI [33]. Moradi et al. (2018) synthesized amino-functionalized MSNs, which significantly enhanced interaction with imidacloprid molecules, delivering a maximum adsorption capacity of 10 mg/g at 60 mg/L imidacloprid [34]. The application of MSNs to efficiently carry active compounds is recognized as a viable means of sustainable pest management [35]. Fig. S5 showed the controlled release profiles of imidacloprid loaded into MSNs over a two-day period. The initial burst release occurred within the first 2 h, which was attributed to diffusion from the hydrophilic mesopores. A continuous release pattern was observed between 2 and 10 h, reaching 100% release by 12 h. The potent adsorption capacity and continuous release performance further proved that MSNs was an excellent nano pesticide delivery carrier.

### Enhanced insecticidal toxicity of IMI@MSNs@lim@chs applied to *Myzus persicae*

**Fig. 3:**
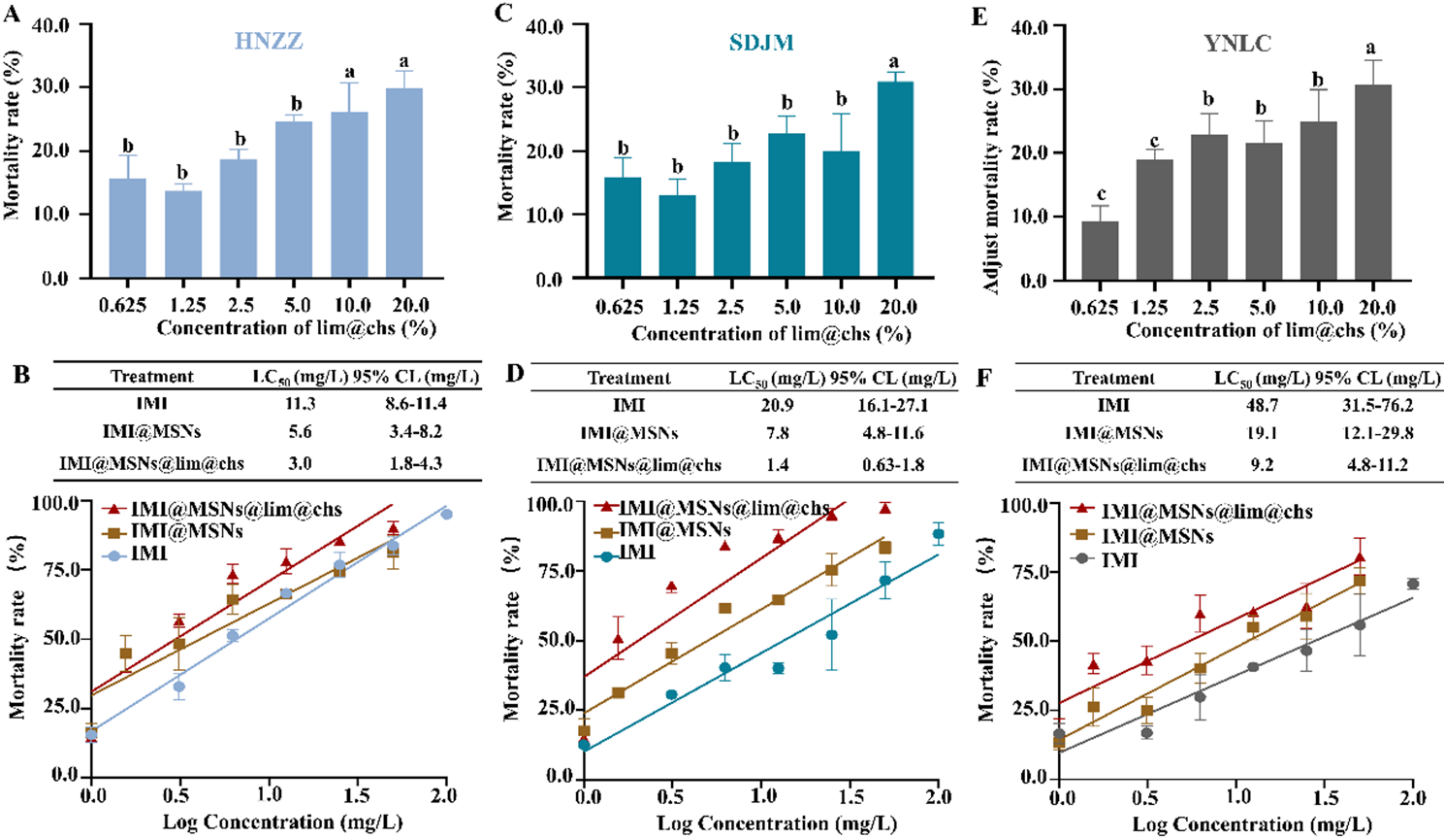
Toxicity bioassays on *Myzus persicae* for three field populations. (**A**), (**C**), and (**E**) The mortality rate of *M. persicae* after treatment with lim@chs. The bars with different lower-case letters are statistically significantly different applying a one-way ANOVA test, followed by a Tukey multiple comparison test (*P* < 0.05). (**B**), (**D**), and (**F**) The analysis of LC_50_ and mortality rate on *M. persicae* after treated with IMI, IMI@MSNs and IMI@MSNs@lim@chs

The toxicity of IMI, and the synergisms of MSNs to IMI were also detected. The results showed that the LC_50_ value of IMI to HNZZ, SDJM, and YNLC field populations was 11.3 mg/L, 20.9 mg/L, and 48.7 mg/L, respectively. From Fig. 3D and F, the LC_50_ value of IMI@MSNs to these three populations was 50.4%, 62.7%, and 60.7% lower than that of IMI, respectively. This indicates that IMI@MSNs had significantly increased toxicity than that of IMI. The MSNs-mediated pesticide delivery system enhanced the pesticide targeted delivery and exhibits significant potential in sustainable pest management [36]. Bilal et al. (2020) have developed carbon dot-embedded fluorescent MSNs, which demonstrated efficient indoxacarb delivery and enhanced its toxicity to *Plutella xylostella* by 1.5-fold [37]. Moreover, Zong et al. (2025) reported that the use of MSNs to deliver chlorantraniliprole, reducing the LC_50_ of chlorantraniliprole to *Spodoptera frugiperda* by over 89%, which was ascribed to a high absorption capacity [38]. 

In the case of lim@chs (20% limonene) toxicity to *M. persicae*, the mortality rate of HNZZ, SDJM, and YNLC field populations reached 29.6%, 31.2%, and 30.6%, respectively (Fig. 3A and C). The IMI@MSNs@lim@chs showed a greater degree of insecticidal toxicity when compared with IMI and IMI@MSNs, representing increases by a factor of 3.8, 14.9, and 5.3, respectively. Our results establish the potency of lim@chs adjuvants in enhancing the insecticide toxicity of imidacloprid to *M. persicae*.

### Inhibition of *Myzus persicae* gut microbiota by lim@chs

**Fig. 4:**
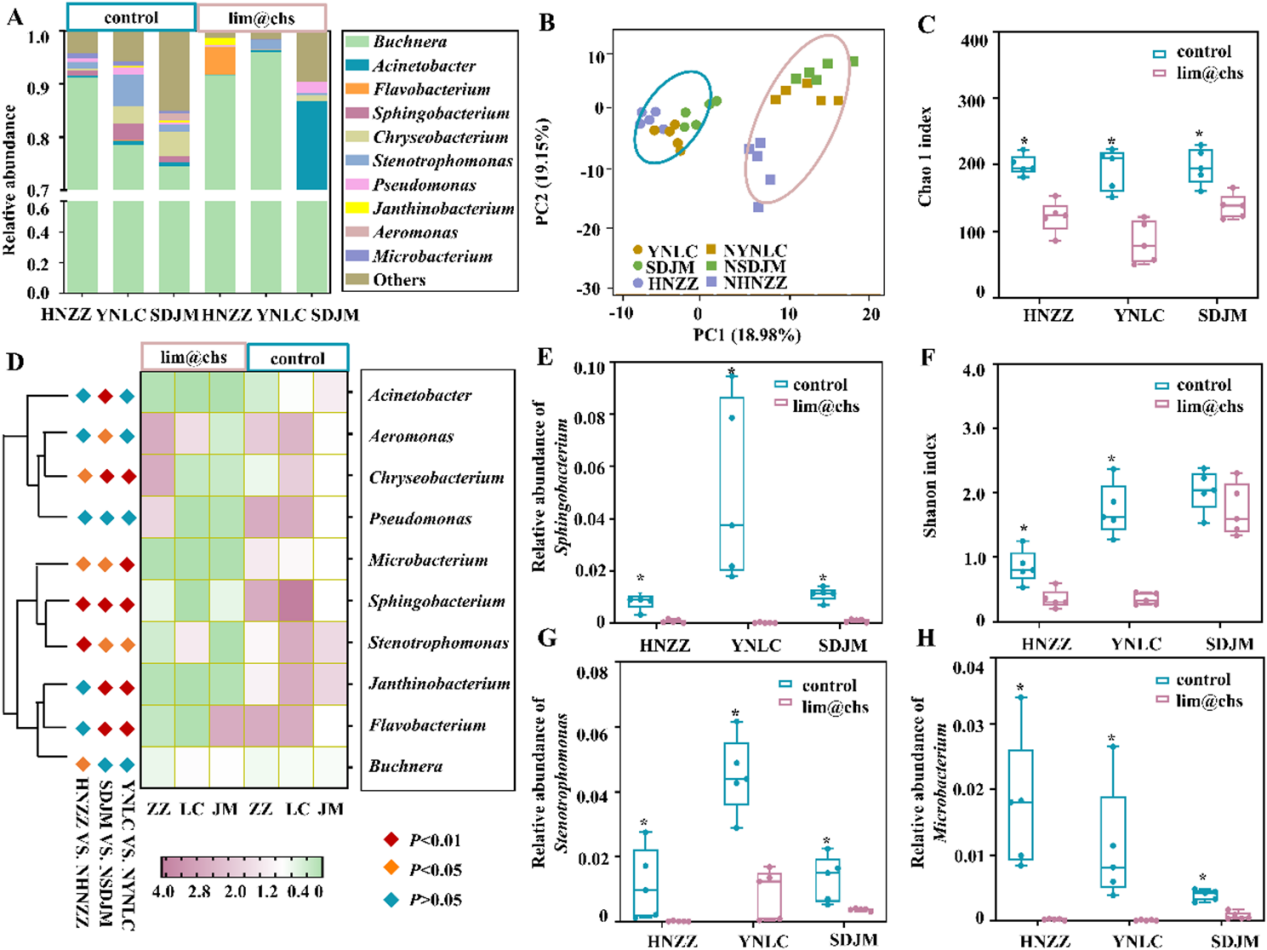
The impact of lim@chs treatment on the gut microbiota of *Myzus persicae.* (**A**) Top ten most abundant gut bacteria at the genus level. (**B**) Heatmap showing changes in the top ten gut symbionts at the genus level following treatment with lim@chs. (**C**) Principal component analysis (PCA) illustrating the dissimilarity in gut microbial composition. (**D**) and (**E**) Shannon and Chao 1 indices used to assess gut symbiont diversity and richness, respectively (Student *t*-test). (**F**) and (**G**) Relative abundance of *Sphingomonas*, *Stenotrophomonas*, and *Microbacterium* (Student *t*-test, “*” *p* < 0.05, FDR corrected)

Limonene has demonstrated effectiveness in inhibiting bacterial growth [39]. Deprivation of gut microbiota suppresses the detoxification capabilities of insects [9]. In order to access the synergism effect in the application of lim@chs and IMI, alterations to gut microbiota in *M. persicae* were assessed following exposure to lim@chs. At the genus level, the abundance of top ten gut microbiota in *M. persica* was shifted following lim@chs treatment (Fig. 4A). A principal component analysis (PCA) has shown that the three field groups were separated from the antibiotic-treated groups, establishing the altered gut microorganism community composition and structure of *M. persica* treated by lim@chs (Fig. 4B). Shannon index and Chao1 index analysis has determined that lim@chs treatment resulted in a significantly decreased gut symbionts diversity and species richness in these three field populations (Fig. 4C and F). Based on the heatmap and histograms, the relative abundance of *Stenotrophomonas*, *Sphingomonas* and *Microbacterium* in the three-field populations was significantly reduced due to lim@chs treatment (Fig. 4D and H). The analysis has demonstrated that lim@chs exhibits potent antibacterial activity with respect to these three genera of *M. persica* gut microorganisms.

**Fig. 5:**
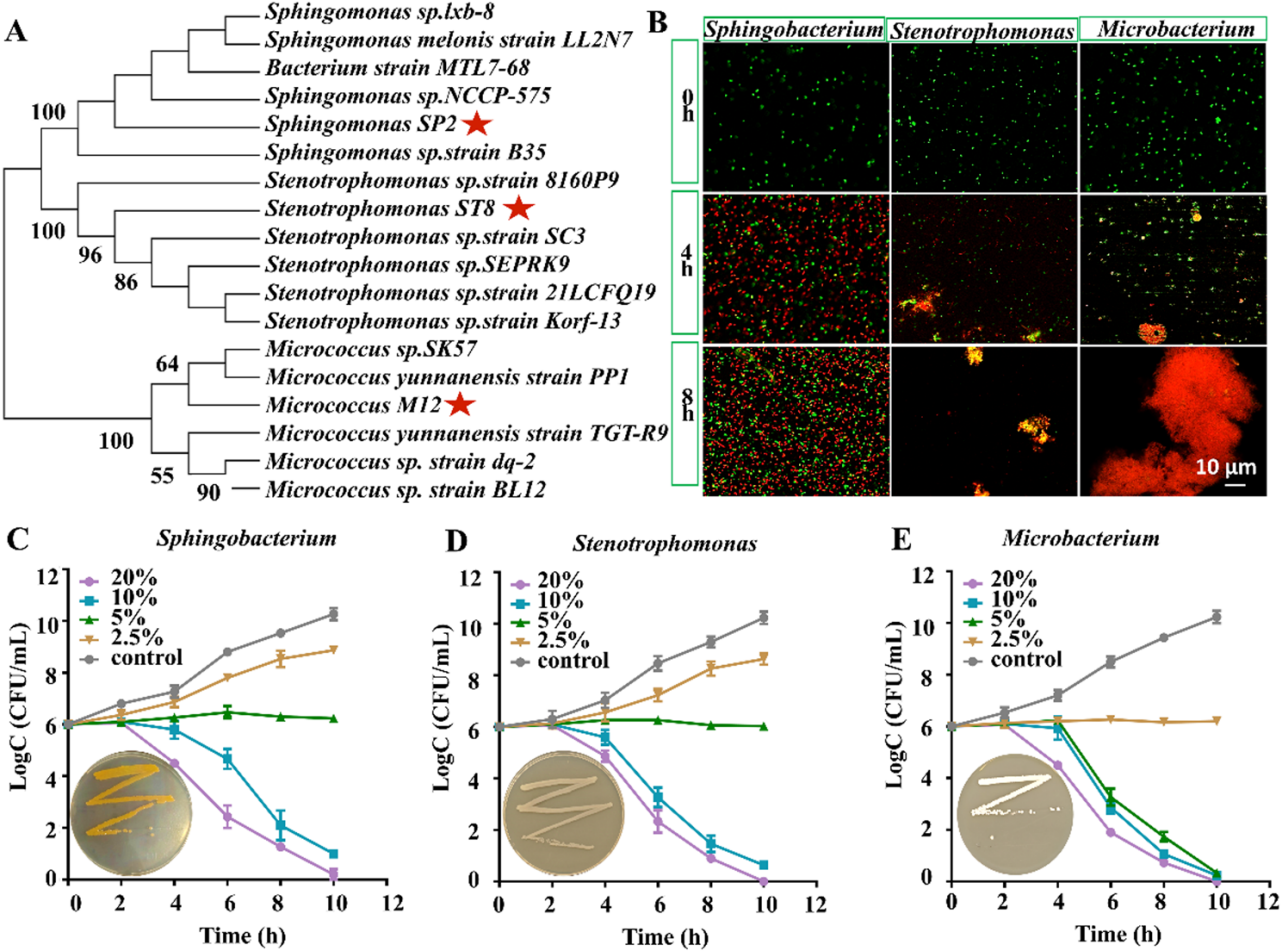
Antibacterial activity of lim@chs with respect to the gut symbiont of *Myzus persicae.* (**A**) Phylogenetic relationships of the symbiotic *Sphingomonas*, *Stenotrophomonas*, and *Microbacterium* strain. The red star denotes the gut symbiont strain isolated from *M. persicae.* (**B**) Gut symbiont analysis following treatment with lim@chs. The green (red) fluorescence indicates viable (dead) bacteria. (**C**)-(**E**) The minimum inhibitory concentration (MIC) of lim@chs to *Sphingomonas*, *Stenotrophomonas*, and *Microbacterium*

Based on the results of 16 S rRNA analysis, the abundance of *Stenotrophomonas*, *Sphingomonas* and *Microbacterium* was significantly inhibited by lim@chs. The *Stenotrophomonas* (16 S rRNA accession No.: PX397422), *Sphingomonas* (PX406541), and *Microbacterium* (PX397418) gut microbiota were isolated, and a phylogenetic tree was constructed (Fig. 5A). The survival of these three genera of gut microorganisms was monitored following lim@chs treatment. The results indicate a time-dependent enhancement of the antibacterial effect (Fig. 5B). To evaluate the minimum inhibitory concentration (MIC) of lim@chs for *Stenotrophomonas*, *Sphingomonas*, and *Microbacterium*, MIC values of the standard antibiotic tetracycline against these three genera of gut microbiota was analysed and presented in Fig. S6. The results show that tetracycline exhibited a consistent MIC of 6.25 mg/L against all three tested bacterial strains. The MIC of lim@chs for the *Stenotrophomonas*, *Sphingomonas* and *Microbacterium* is 5%, 5% and 2.5%, respectively (Fig. 5C and E). This response demonstrates that lim@chs is effective in inhibiting these three genera of gut microbiota. It is known that limonene and chitosan can interact with and disrupt bacterial cell membranes, resulting in inhibited bacterial growth. Indeed, encapsulation of limonene within nanoemulsions can increase antibacterial activity by improving absorption and bioavailability [40, 41]. 

### Suppressed P450 detoxification capability of *Myzus persicae* due to lim@chs-induced gut microbiota deficiency

**Fig. 6:**
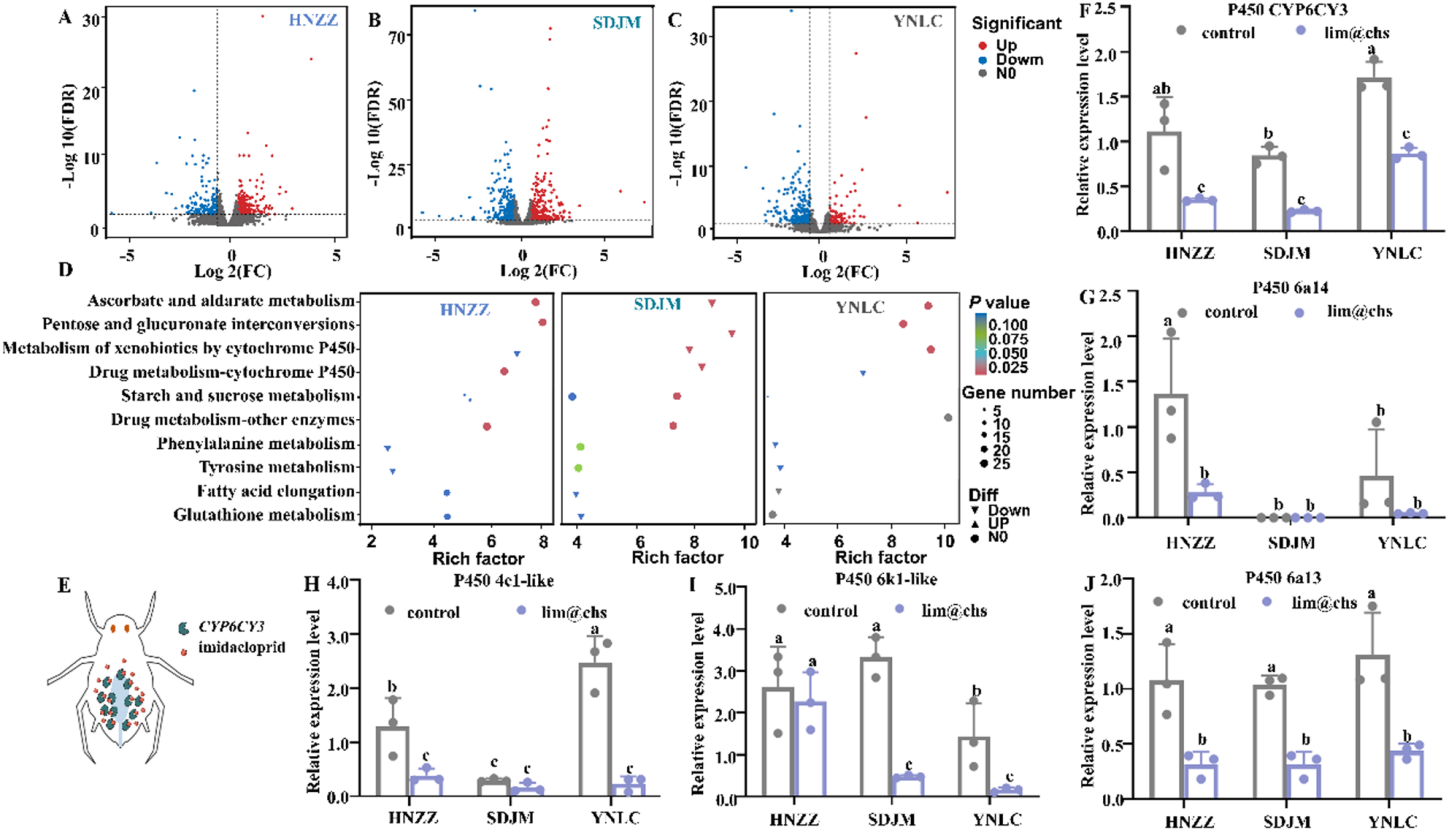
Transcriptional profiles and the relative expression of P450 genes in *Myzus persica* after treatment with lim@chs. (**A**-**C**) Volcano plots showing the upregulated and downregulated genes in three field populations of *M. persicae*. (**D**) The regulated KEGG pathways in *M. persica*. (**E**) The resistance mechanism of *CYP6CY3* in *M. persica* to imidacloprid. (**F**-**J**) The supressed P450 genes in *M. persica*. The bars with different lowercase letters are significantly different according to one-way ANOVA, followed by Tukey’s multiple comparison test (*P* < 0.05)

Transcriptome analysis was conducted on *M. persica* to establish the detoxification capacity. The relative expression of an appreciable number of genes was significantly altered following treatment with lim@chs. In the case of HNZZ, SDJM, and YNLC field populations, 277, 330, and 108 genes were upregulated and 171, 281, and 242 genes were downregulated, respectively (Fig. 6A C). The Kyoto Encyclopedia of Genes and Genomes (KEEG) pathways were analysed for the three populations after lim@chs treatment. The pathways related to detoxification metabolism were downregulated, including metabolism of xenobiotics by cytochrome P450, and drug metabolism-cytochrome P450 (Fig. 6D). Furthermore, the expression of *CYP6CY3*, *cytochrome P450 6a13*, *cytochrome P450 6a14*,* cytochrome P450 4c1-lik*e, and *cytochrome P450 6k1-like* was significantly downregulated in *M. persica* following lim@chs treatment (Fig. 6F and G). Zhan et al. (2018) have reported that antibiotic consumption disrupts the gut microbiota and reduces the expression of P450 genes in rats [7]. Moreover, depletion of the gut microbiota in honey bees led to a decrease in the expression of P450 genes [8]. The studies confirm that *CYP6CY3* plays a crucial role in the resistance of *M. persica* to IMI, where RNA interference-mediated knockdown of *CYP6CY3* expression significantly increases the sensitivity of *M. persica* to IMI [42, 43]. The *CYP6CY3* enhances the resistance of *M. persica* to IMI by metabolizing IMI into less toxic metabolites, where the over-expression of *CYP6CY3* in *M. persica* contributes to an increased metabolic resistance to IMI (Fig. 6E) [44]. In addition, after re-introduction gut bacteria, the *CYP6CY3* expression in YNLC population of lim@chs treated*-M. persicae* showed a significant increase (Fig. S7). These results further confirmed that the deficiency of gut microbiota suppresses *CYP6CY3* expression, which in turn contributes to the significantly increased susceptibility of *M. persicae* to IMI.

### Impaired cuticle by lim@chs to enhance imidacloprid penetration

**Fig. 7:**
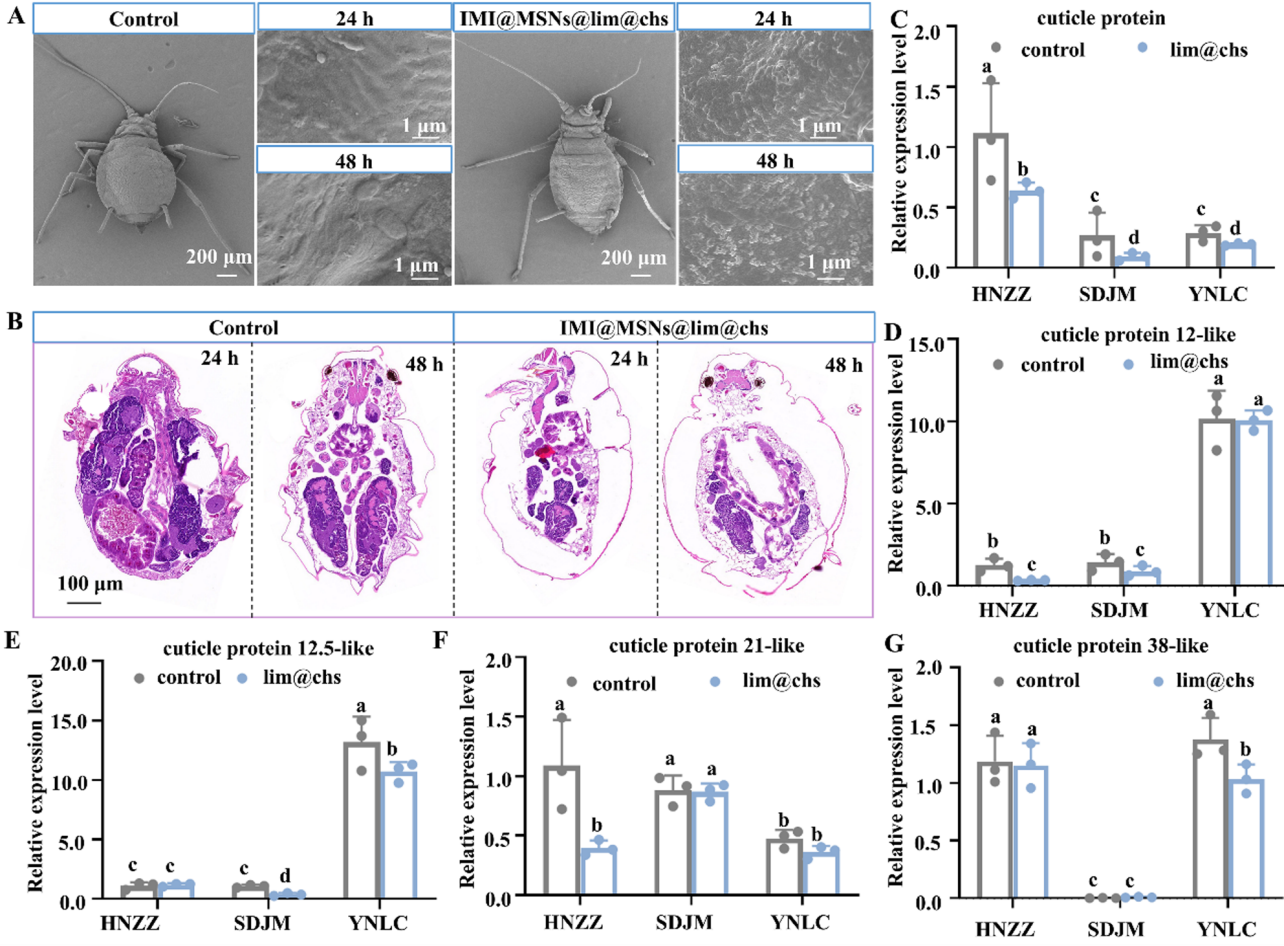
The cuticle observation and cuticle proteins analysis in *Myzus persicae* after treatment with lim@chs. (**A**-**B**) SEM and paraffin sections images showed the impaired cuticle of *M. persica*. (**C**-**G**) The supressed cuticle protein genes in *M. persica*. The bars with different lowercase letters are significantly different according to one-way ANOVA, followed by Tukey’s multiple comparison test (*P* < 0.05)

**Fig. 8:**
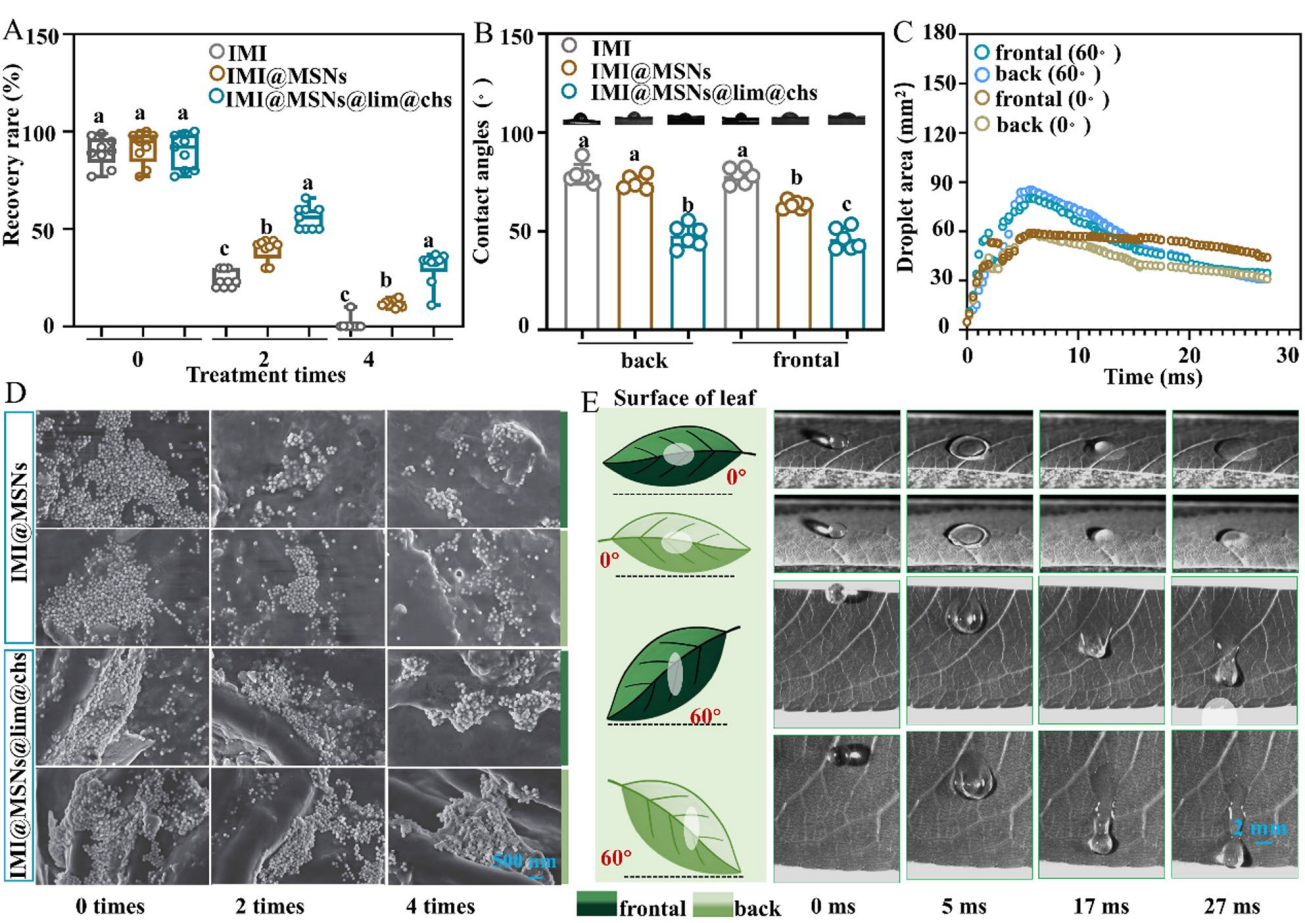
Adhesion, surface wettability and deposition efficiency of the IMI@MSNs@lim@chs nanosystem. (**A**) The recovery rate of imidacloprid after washing. (**B**) Contact angles of different solutions on peach foliar surfaces. (**D**) SEM images of different samples on peach leaves before and after washing. (**C**, **E**) Variation of the IMI@MSNs@lim@chs droplet area as a function of time and the droplet impact dynamics on the peach leaves. The bars with different lower-case letters are statistically significantly different applying a one-way ANOVA test, followed by a Tukey multiple comparison test (*P* < 0.05)

The SEM analysis has revealed that the cuticle of *M. persica* is smooth and flat, but exhibited a rough and uneven surface after treatment with lim@chs (Fig. 7A). Moreover, the longitudinal section of the insect body shows the separation of the epidermis and internal tissues after treatment with lim@chs (Fig. 7B). The relative expression level measurements have revealed a suppression of 7-like, 12-like, 12.5-like, 21-like and 38-like cuticle proteins due to the lim@chs treatment (Fig. 7C and G). The insect cuticle consists of multiple layers, where the outermost layer is typically coated with wax and serves as the primary barrier against the penetration of external compounds [45]. This wax layer is composed of long-chain fatty acids and their derivatives, such as aldehydes, alcohols, ketones, and esters [46]. lim@chs can readily penetrate the insect epidermis, due to its hydrophobicity and lipophilicity (Fig. 6N), which would benefit to improve the penetration rate of imidacloprid. To further assess whether the MSNs and lim@chs adjuvants enhanced the penetration efficiency of imidacloprid into aphids, HPLC-MS was analysis was performed. The amount of imidacloprid delivered into the aphids was quantified based on the imidacloprid standard curve (Fig. S8) and the recovery efficiency (Fig. S9). As shown in Fig. 8A, the imidacloprid content in IMI@MSNs-treated *M. persicae* was 0.34 ng/mg, which was 1.4-fold higher than that in imidacloprid-treated *A. gossypii* (0.24 ng/mg). Consistent with expectations, lim@chs@IMI@MSNs-treated *M. persicae* showed a markedly increased delivery of imidacloprid (0.47 ng/mg), corresponding to 1.95 times the level observed in imidacloprid-treated *M. persicae*. The results indicate that the MSN-based delivery system enhanced the penetration efficiency of imidacloprid. Moreover, the lim@chs adjuvant treatment exhibited a significant synergistic effect with IMI@MSNs by disrupting the cuticle of *M. persicae*, thereby improving pesticide delivery and ultimately contributing to potent insecticidal activity against *M. persicae*.

### Improved adhesion, wettability and deposition efficiency of IMI@MSNs@lim@chs

Improved adhesion, wettability properties and foliar deposition efficiency can enhance pesticide utilization and targeting, facilitating effective field applications. The foliar adhesion of IMI, IMI@MSNs, and IMI@MSNs@lim@chs on peach leaf surfaces was determined by simulating rainwater leaching. The IMI recovery rate was determined according to the stand curve and recovery efficiency of IMI (Fig. S10). As shown in Fig. 8A, the IMI recovery rate with increasing washing time was significantly reduced for the IMI, IMI@MSNs, and IMI@MSNs@lim@chs treatment groups. A near complete IMI removal after four washes was observed for the IMI treatment group, whereas 11.6% and 30.6% of the IMI was removed from the IMI@MSNs and IMI@MSNs@lim@chs treated samples. The SEM analysis provides evidence of more residual IMI@MSNs@lim@chs nanoparticles on the peach leaves when compared with the IMI@MSNs treatment group after two and four washes (Fig. 8D). The porous MSNs adhere effectively in the folds of the leaf surface [47]. Following rainwater leaching, the IMI on the MSN nanocarrier is more resistant to removal. The foliar wettability of IMI, IMI@MSNs, and IMI@MSNs@lim@chs on the peach foliar surfaces was examined by contact angle measurement (Fig. 8B). The contact angles for IMI and IMI@MSNs on the back (frontal) peach leaves are approximately 78.7 (78.0) and 74.7 (63.5), respectively. The contact angle for IMI@MSNs@lim@chs was significantly lower (47.7^◦^ (46.0^◦^)), indicting enhanced wettability that can be attributed to lim@chs. The leaf surface is covered by a waxy layer composed of lipid-soluble compounds. As limonene exhibits lipophilic characteristics [48, 49], it can interact with the waxes *via* hydrogen bonding, which modifies the structure or viscosity [50]. Consequently, the bond between the peach leaves and IMI@MSNs@lim@chs is strengthened, which further enhances wettability and adhesion.

The peach leaves were used as a test surface to establish the foliar deposition efficiency of IMI@MSNs@lim@chs. The images taken using a high-speed camera demonstrate that the droplets undergo (i) impact, (ii) spreading, and (iii) retraction (Fig. 8E). The evolution of the droplet diameter on peach leaves, both back and front, when IMI@MSNs@lim@chs impacts the leaves perpendicularly and at a 60° angle is shown in Fig. 8C. During initial impact and expansion, inertial forces dominate, with droplets (≈ 2 mm) splitting at high velocity. The impact causes the droplets to reach maximum diameter at ≈ 5 ms for both flat and 60° tilted peach leaves. This is followed by a retraction stage where viscous and capillary forces, driven by surface tension, govern wetting and adhesion [51]. Droplet retention on foliage surfaces is hindered by the waxy covering, which is characterized by a “hairy”, curved, or rough morphology [52]. These microstructural features result in an asymmetric impact, which contributes to greater droplet fragmentation and lower deposition efficiency [53]. This suggests that droplet rebound is negligible, resulting in significant deposition efficiency on the leaves, which is critical for improving pesticide utilization and minimizing application volumes.

### IMI@MSNs@lim@chs exhibited enhanced insecticidal activity and reduced environmental toxicity

**Fig. 9:**
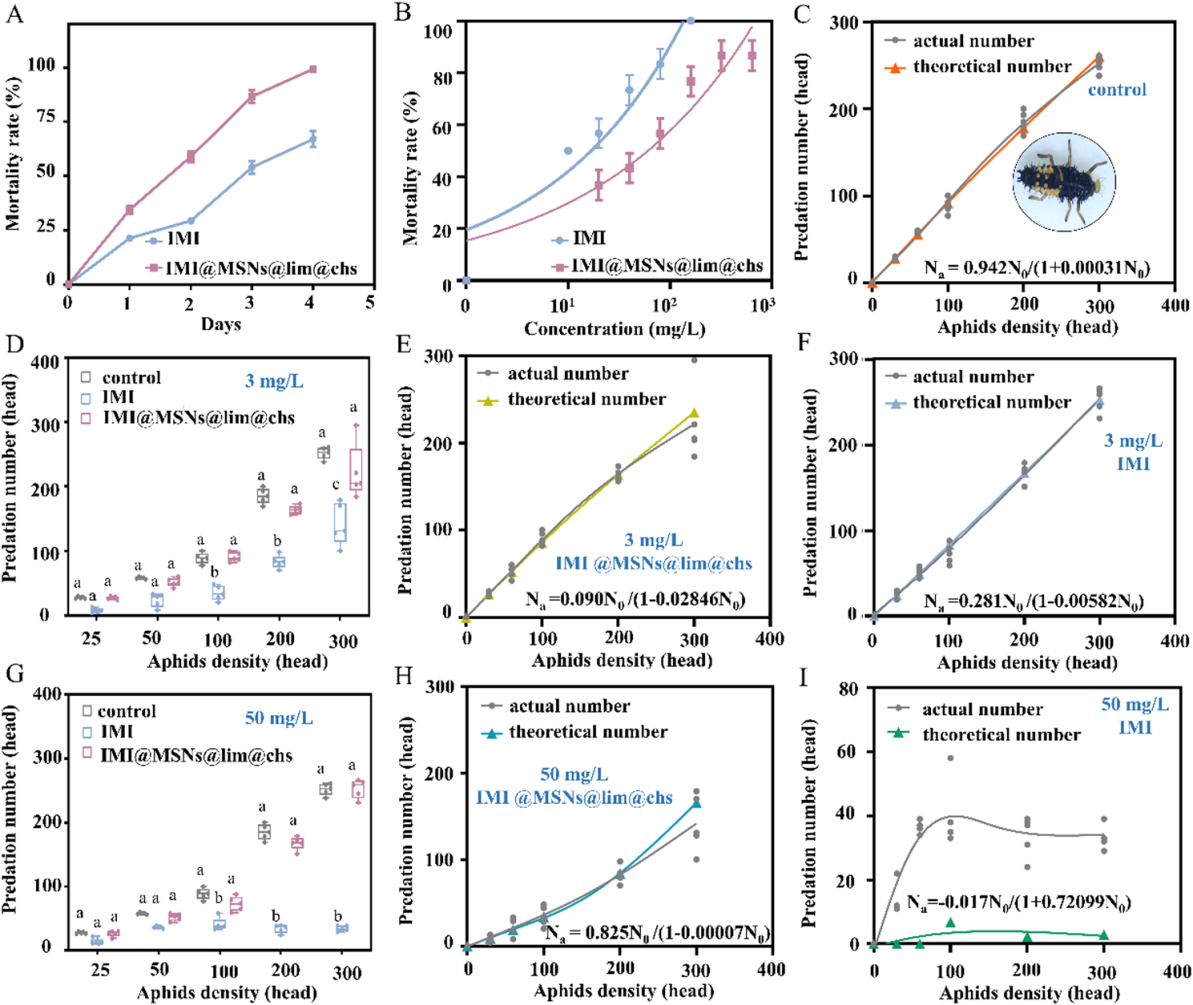
Insecticidal activity with respect to *Myzus persicae* and toxicity to *Coccinella septempunctata* evaluated for IMI@MSNs@lim@chs. (**A**) Mortality of *M. persicae* treated with IMI or IMI@MSNs@lim@chs. (**B**) Mortality rate curve for *C. septempunctata* following treatment with IMI or IMI@MSNs@lim@chs. (**D**) and (**G**) Number of aphids consumed by *C. septempunctata* after exposure to 3 mg/L and 50 mg/L of IMI or IMI@MSNs@lim@chs. (**C**), (**E**), (**F**), (**H**), and (**I**) Predator functional response equations for *C. septempunctata* derived from the control group and from groups treated with 3 mg/L and 50 mg/L of IMI or IMI@MSNs@lim@chs

 The insecticidal activity of IMI@MSNs@lim@chs to *M. persicae* was evaluated in greenhouse tests. The mortalities of *M. persicae* reached 100% after treatment with IMI@MSNs@lim@chs for four days (Fig. 9A), significantly higher than IMI treatment (68.9%), indicating enhanced control efficacity of IMI@MSNs@lim@chs. As shown in Fig. 9B, the toxicity of IMI@MSNs@lim@chs to *Coccinella septempunctata* was significantly reduced when compared with IMI. The LC_50_ value of IMI@MSNs@lim@chs for *C. septempunctata* was 46.5 mg/L, which is a factor of 3.6 times higher than IMI (12.9 mg/L) (Table S5). The active ingredient of IMI is limited in IMI@MSNs@lim@chs, resulting in a reduction toxicity to *C. septempunctata*. Papanikolaou et al. (2018) have demonstrated that a nano-formulated pyrethrin improved insecticidal activity for the target *Aphis gossypii*, whereas the toxicity to non-target *C. septempunctata* predator was reduced [54]. Farhan and co-workers (2024) established that nano-formulations of chlorpyrifos and pyrethroids delivered enhanced mortality rates for mosquitoes with reduced toxicity in the case of *C. septempunctata.* [55] The predator functional response equation indicates that the predation capacity of *C. septempunctata* increased with aphid density (Fig. 9C). When the aphid density increased to 100, the number of aphids consumed by *C. septempunctata* treated with 3 mg/L and 50 mg/L IMI was significantly reduced relative to the control group (Fig. 9D and E). Pesticide exposure can negatively affect the predation capability of natural enemy insects [56]. For example, the predation capacity of *Spodoptera frugiperda* was significantly reduced at a sufficient density of *Podisus nigrispinus* following exposure to imidacloprid [57]. The predator functional response analysis has confirmed that LC_10_ and LC_50_ doses of IMI suppressed the predation capability of *C. septempunctata* (Fig. 9F and I). In contrast, the predation capacity of *C. septempunctata* was not significantly inhibited by 3 mg/L or 50 mg/L IMI@MSNs@lim@chs treatment (Fig. 9E and H). Wu et al. (2024) have reported that star cationic polymer (SPc)-loaded TTP (tetraniliprole) showed significantly improved toxicity to *Spodoptera litura*, but did not exhibit any negative effect on predators [58]. Pesticides have inherent environmental risks, and the nano-pesticides was impossible absolutely ecological safety. These findings suggest that nano-pesticides have decreased impact on predators and are safer for the environment when compared with conventional pesticides alone.

## Conclusions

In summary, we developed a multifunctional nanosystem (IMI@MSNs@lim@chs) by dispersing IMI-loaded mesoporous silica nanospheres in limonene–chitosan Pickering emulsions. The IMI@MSNs complex significantly increased the toxicity of IMI to *Myzus persicae*, due to an efficient pesticide absorption and delivery. The hybrid platform IMI@MSNs@lim@chs achieved the highest efficacy against *M. persicae*. Mechanistic studies have revealed that lim@chs serves to disrupt gut symbionts and suppress P450-mediated detoxification, while also impairing the insect cuticle to promote epidermal penetration. In parallel, the porous architecture of MSNs facilitated adhesion, and the lipophilic nature of lim@chs improved wettability on hydrophobic leaves, together enhancing foliar deposition, targeting, and persistence. Importantly, IMI@MSNs@lim@chs exhibits reduced toxicity and negligible sublethal effects for *Coccinella septempunctata* when compared with IMI alone, demonstrating environmental compatibility. Nonetheless, it is crucial to recognize the limitations that may impact practical field applications, including potential photodegradation, the volatility of limonene, which may affect efficacy of IMI@MSNs@lim@chs. Besides, the potential accumulation of nanoparticles in soil warrants attention. These factors underscore the need for comprehensive long-term environmental impact assessments of the IMI@MSNs@lim@chs system.

In summary, despite limitations, these findings demonstrate the power of combining porous nanocarriers with microbiota-targeting Pickering emulsions to achieve synergistic pesticide delivery. This strategy offers a promising path toward more efficient, selective, and sustainable pest management solutions. 

## Supplementary Information

Supplementary Material 1

## Data Availability

The data that support the findings of this study are available in the supplementary material of this article.
