# Supplementary material for: Coupled mesoporous silica nanoparticles and limonene–chitosan Pickering emulsions: enhanced insecticidal delivery and selectivity
Source: J Nanobiotechnology. 2026 Mar 19;24:395. doi: 10.1186/s12951-026-04172-0 (PMC13122875; doi:10.1186/s12951-026-04172-0)
Supplement: Supplementary file 1 — Supplementary Material 1 [file 12951_2026_4172_MOESM1_ESM.docx]

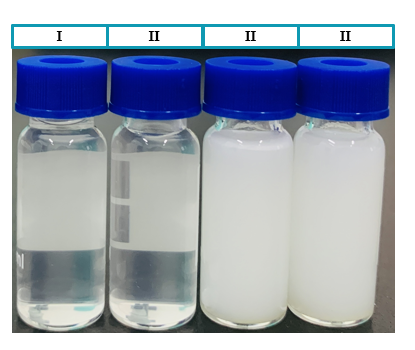


**Fig. S1**. The image of I (IMI), II (IMI@MSNs), III (chs@lim), and (IV) IMI@MSNs@chs@lim.


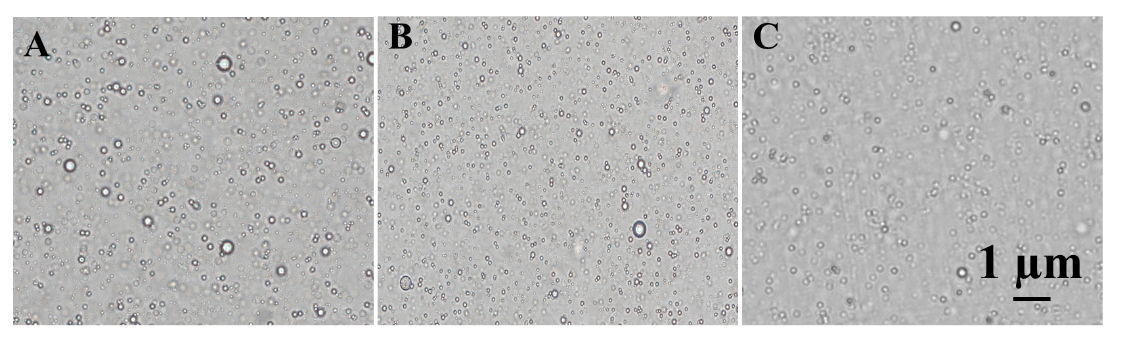


**Fig. S2**. Optical microscopy images of lim@chs with different storge times. (A) 1 day, (B) 10 days, (C) 20 days.


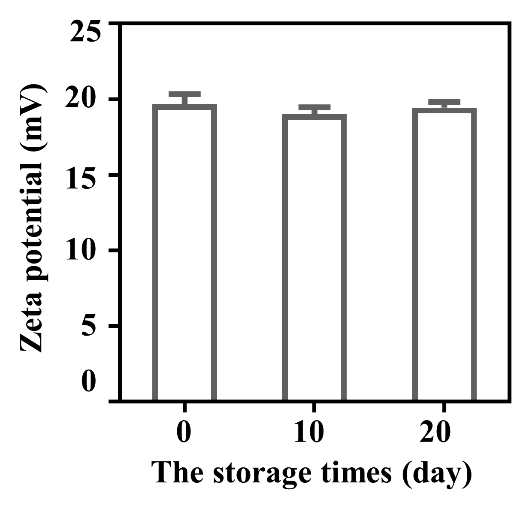


**Fig. S3**. Zeta potential data of lim@chs with different storge times.


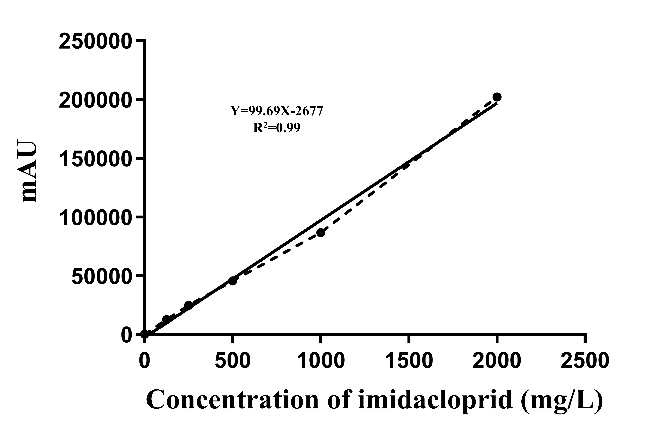


**Fig. S4**. The standard curve of imidacloprid on HPLC.


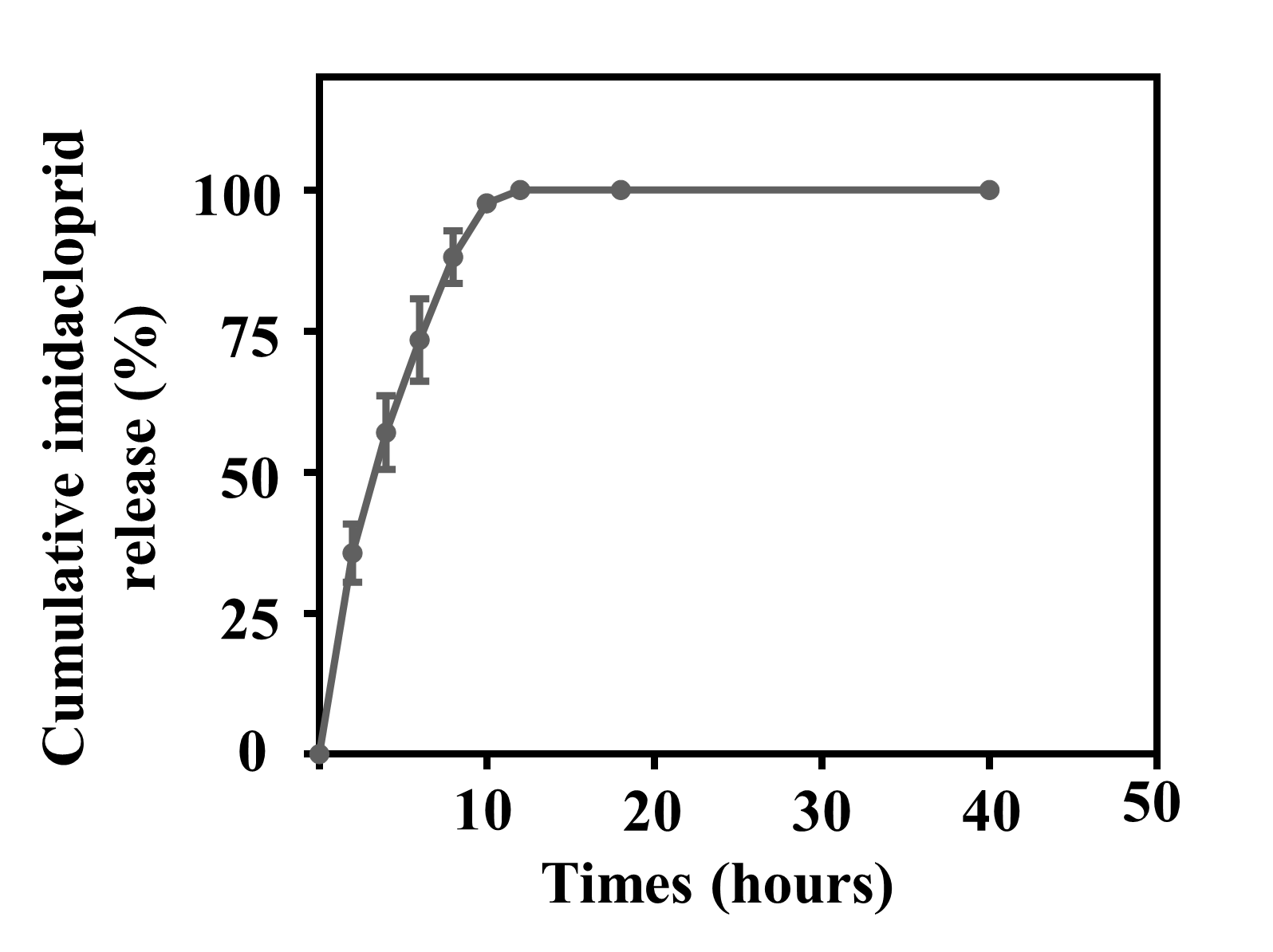


**Fig. S5**. Cumulative release of imidacloprid from MSNs.

**
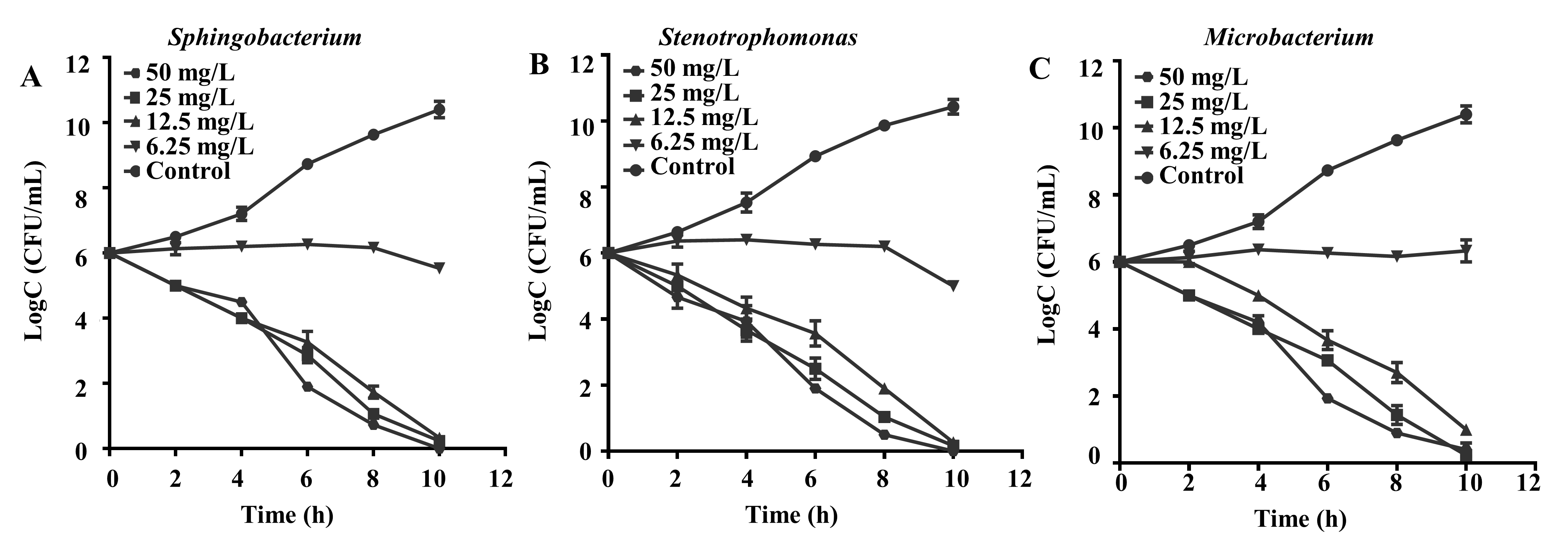
**

**Fig. S6**. The minimum inhibitory concentration (MIC) of tetracycline to *Sphingomonas* (A), *Stenotrophomonas* (B), and *Microbacterium* (C)*.*

**
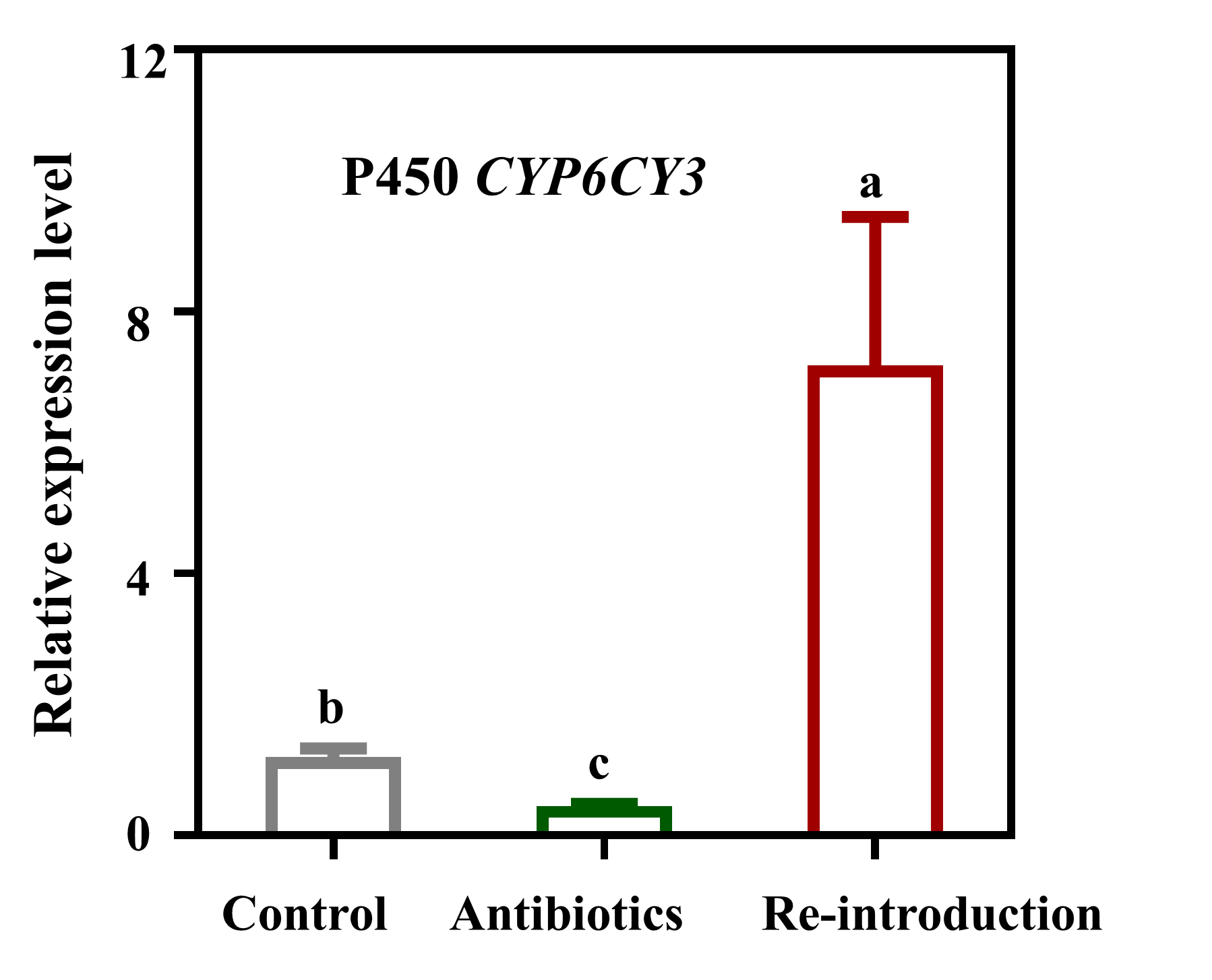
**

**Fig. S7**. The *CYP6CY3* expression in YNLC population of *Myzus persicae.* The bars with different lowercase letters are significantly different according to one-way ANOVA, followed by Tukey's multiple comparison test (*P*< 0.05).


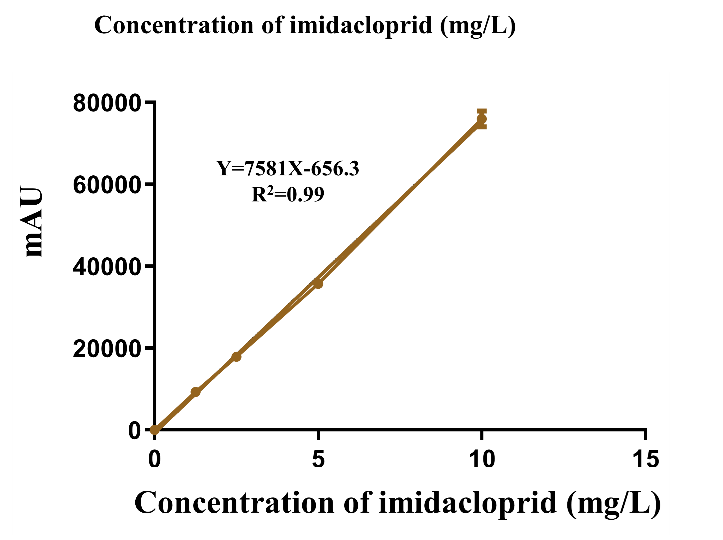


**Fig. S8**. The standard curve of imidacloprid on HPLC-MS.


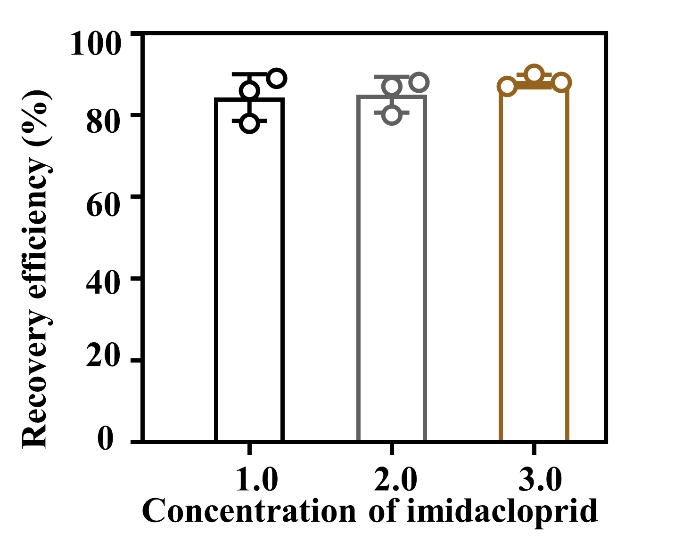


**Fig. S9**. The recovery efficiency of imidacloprid from the homogenate of *Myzus persicae.*


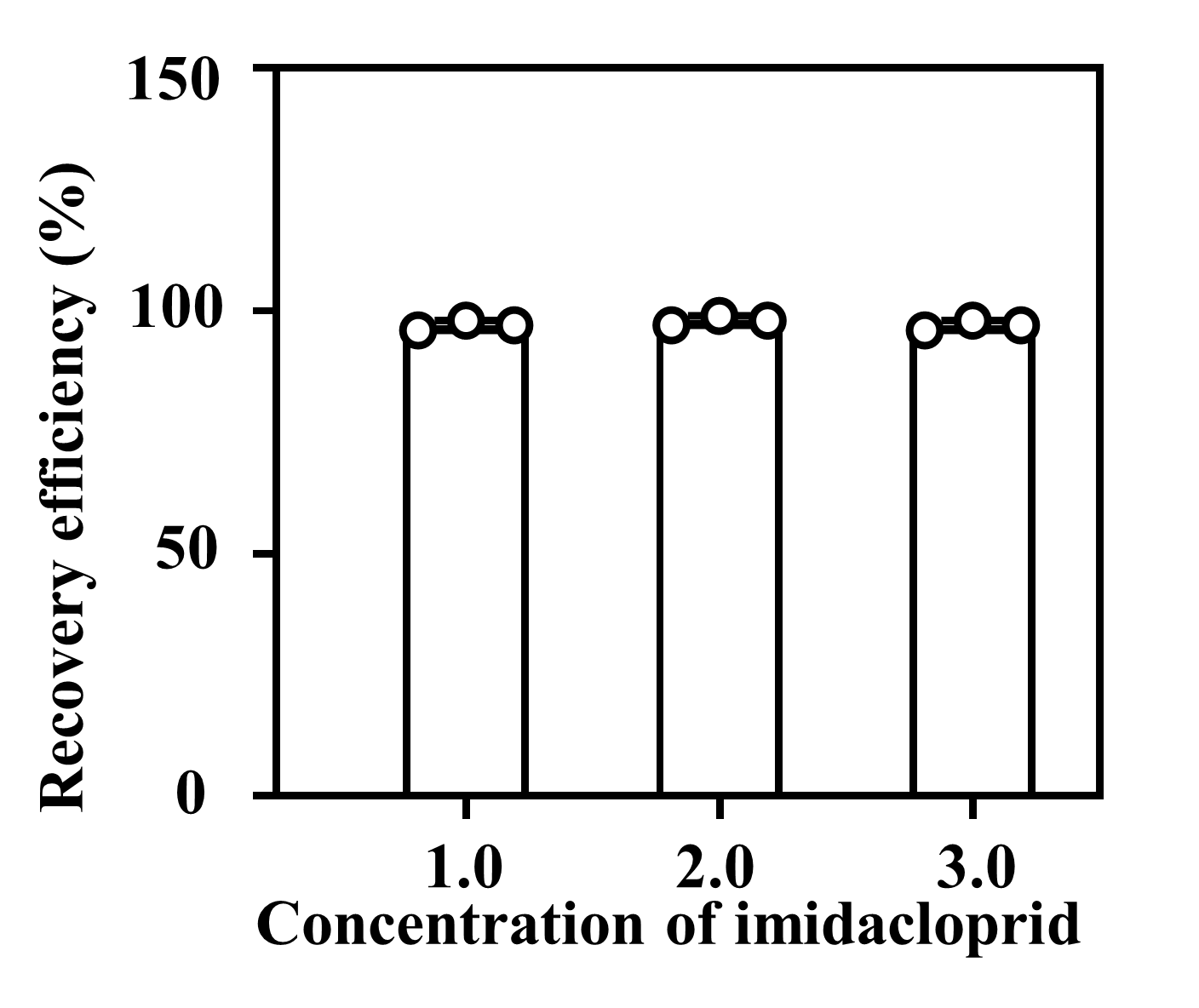


**Fig. S10**. The recovery efficiency of imidacloprid for leaching experiment.

Table S1 The information of genes

| Gene-id | Gene name | NR Nation |
| --- | --- | --- |
| gene9605 | *P4504C1-like* | [PREDICTED: *Myzus persicae cytochrome P450 4C1-like* (LOC111034224), mRNA](https://blast.ncbi.nlm.nih.gov/Blast.cgi#alnHdr_1229900590) |
| gene4273 | *P4506a14* | [PREDICTED: *Myzus persicae probable cytochrome P450 6a14* (LOC111028277), mRNA](https://blast.ncbi.nlm.nih.gov/Blast.cgi#alnHdr_1229884737) |
| gene7532 | *P4506k1-like* | [PREDICTED: *Myzus persicae cytochrome P450 6k1-like* (LOC111031899), partial mRNA](https://blast.ncbi.nlm.nih.gov/Blast.cgi#alnHdr_1229894348) |
| gene3895 | *CYP6CY3* | [*Myzus persicae clone 4255A cytochrome P450 CYP6CY3 (CYP6CY3)* mRNA, complete cds](https://blast.ncbi.nlm.nih.gov/Blast.cgi#alnHdr_563407370) |
| gene5869 | *P4506a13* | PREDICTED: *Myzus persicae probable cytochrome P450 6a13* (LOC111030036), transcript variant X1, mRNA |
| gene15901 | *Cuticle protein 12-like* | [PREDICTED: *Myzus persicae flexible cuticle protein* 12-like (LOC111041246), mRNA](https://blast.ncbi.nlm.nih.gov/Blast.cgi#alnHdr_1230324398) |
| gene16829 | *Cuticle protein 12.5-like* | [PREDICTED: *Myzus persicae cuticle protein 12.5-like* (LOC111042286), mRNA](https://blast.ncbi.nlm.nih.gov/Blast.cgi#alnHdr_1230417450) |
| gene13931 | *Cuticle protein 19-like* | [PREDICTED: *Myzus persicae cuticle protein 19-like* (LOC111039064), mRNA](https://blast.ncbi.nlm.nih.gov/Blast.cgi#alnHdr_1230124773) |
| gene8018 | *Cuticle protein 38-like* | [PREDICTED: *Myzus persicae cuticle protein 38-like* (LOC111032404), mRNA](https://blast.ncbi.nlm.nih.gov/Blast.cgi#alnHdr_1229895640) |
| gene13936 | *Cuticle protein 21-like* | [PREDICTED: *Myzus persicae cuticle protein 21-like* (LOC111039055), mRNA](https://blast.ncbi.nlm.nih.gov/Blast.cgi#alnHdr_1230124170) |
| gene13929 | *Cuticle protein 7-like* | [PREDICTED: *Myzus persicae cuticle protein 7-like* (LOC111039057), mRNA](https://blast.ncbi.nlm.nih.gov/Blast.cgi#alnHdr_1230124628) |

Table S2 Primers used in this study for qPCR

| Gene-id | Prime name | Sequence of primers |
| --- | --- | --- |
| 7532 | *P450 6k1-like-*F | CTTGCGCGTTTGGTCTGAAA |
| 7532 | *P450 6k1-like-*R | GACGACGTCAGTGAACACCT |
| 3895 | *CYP6CY3-*F | ATCAGGCCGATTCCGTTGTT |
| 3895 | *CYP6CY3-*R | ATTCGCCGCTAAATCCGAGT |
| 4273 | *P450 6a14-*F | TTACAACCAGTTCCCGGACG |
| 4273 | *P450 6a14-*R | GGACTGAGCTTCTGTCGCAT |
| 5869 | *P450 6a13-*F | AAGCTAACGTGCCGCATTTG |
| 5869 | *P450 6a13-*R | AACCCACGGTCTGTGAAGTG |
| 9605 | *P450 4C1-like-*F | CGATCCCGACCGATTTTTGC |
| 9605 | *P450 4C1-like-*R | TGTGTGCTGATGACGATGCT |
| 13929 | *Cuticle protein 7-like-*F | GCGTAAACGATCCACACACC |
| 13929 | *Cuticle protein 7-like-*R | CGGCGTTGAAACCGTTGTAG |
| 13931 | *Cuticle protein 19-like-*F | GTCAAGAGCCAACGCGAATC |
| 13931 | *Cuticle protein 19-like-*R | GCGGAGTAAGCTGGCTTGTA |
| 13936 | *Cuticle protein 21-like-*F | TACAGCGTCAACGACCCATC |
| 13936 | *Cuticle protein 21-like-*R | TTCTTGACCTCGGCGTTGAA |
| 15901 | *Cuticle protein 12-like-*F | TGCTGCGGTGACCACATATT |
| 15901 | *Cuticle protein 12-like-*R | CCAACGTACCTTCCCTGGTC |
| 16829 | *Cuticle protein 12.5-like-*F | TACGGACTGGGTTACGGCTA |
| 16829 | *Cuticle protein 12.5-like-*R | GGGGGATGCGTAAGAGATGG |
| 8018 | *Cuticle protein 38-like-*F | TACGCTAGCTCGTTCACGTC |
| 8018 | *Cuticle protein 38-like-*R | AGTAACCGTAAGGGGAGGCT |
|  | *MpActin-*F | GGTGTCTCACACACAGTGCC |
|  | *MpActin-*R | CGGCGGTGGTGGTGAAGCTG |

Table S3 Homogeneity and normality tests (Levene's and Shapiro-Wilk).

| Analysis | Group | Shapiro-Wilk test for Assumptions of Normality Sig. ^a^ | Levene's test for Equality of Variances Sig. (Base on MEAN)^b^ |
| --- | --- | --- | --- |
| Fig. 1G (IMI@MSNs@lim@chs) | 50 | 0.085 | 0.456 |
|  | 60 | 0.637 |  |
|  | 70 | 0.637 |  |
|  | 80 | 0.637 |  |
| Fig. 1G (lim@chs) | 50 | 1.000 | 1.000 |
|  | 60 | 1.000 |  |
|  | 70 | 1.000 |  |
|  | 80 | 1.000 |  |
| Fig. 2G | [IMI@MSNs@lim@chs](mailto:IMI@MSNs@lim@chs) | 0.599 | 0.989 |
|  | IMI@MSNs | 0.577 |  |
|  | MSNs | 0.307 |  |
| Fig. 3A | 0.625 | 0.671 | 0.057 |
|  | 1.25 | 0.915 |  |
|  | 2.5 | 0.939 |  |
|  | 5.0 | 0.570 |  |
|  | 10.0 | 0.187 |  |
|  | 20.0 | 0.103 |  |
| Fig. 3C | 0.625 | 0.094 | 0.146 |
|  | 1.25 | 0.849 |  |
|  | 2.5 | 0.979 |  |
|  | 5.0 | 0.820 |  |
|  | 10.0 | 0.194 |  |
|  | 20.0 | 0.064 |  |
| Fig. 3E | 0.625 | 0.411 | 0.337 |
|  | 1.25 | 0.595 |  |
|  | 2.5 | 0.900 |  |
|  | 5.0 | 0.648 |  |
|  | 10.0 | 0.226 |  |
|  | 20.0 | 0.567 |  |
| Fig. 4C (HNZZ) | control | 0.719 | 0.655 |
|  | lim@chs | 0.640 |  |
| Fig. 4C (YNLC) | control | 0.256 | 0.928 |
|  | lim@chs | 0.439 |  |
| Fig. 4C (SDJM) | control | 0.905 | 0.364 |
|  | lim@chs | 0.519 |  |
| Fig. 4E (HNZZ) | control | 0.879 | 0.070 |
|  | lim@chs | 0.157 |  |
| Fig. 4E (YNLC) | control | 0.518 | 0.682 |
|  | lim@chs | 0.202 |  |
| Fig. 4E (SDJM) | control | 0.626 | 0.055 |
|  | lim@chs | 0.412 |  |
| Fig. 4F (HNZZ) | control | 0.746 | 0.369 |
|  | lim@chs | 0.177 |  |
| Fig. 4F (YNLC) | control | 0.741 | 0.069 |
|  | lim@chs | 0.312 |  |
| Fig. 4F (SDJM) | control | 0.641 | 0.361 |
|  | lim@chs | 0.532 |  |
| Fig. 4G (HNZZ) | control | 0.281 | 0.177 |
|  | lim@chs | 0.302 |  |
| Fig. 4G (YNLC) | control | 0.333 | 0.101 |
|  | lim@chs | 0.328 |  |
| Fig. 4G (SDJM) | control | 0.283 | 0.186 |
|  | lim@chs | 0.260 |  |
| FiH. 4H (HNZZ) | control | 0.293 | 0.053 |
|  | lim@chs | 0.214 |  |
| FiH. 4H (YNLC) | control | 0.569 | 0.241 |
|  | lim@chs | 0.100 |  |
| FiH. 4H (SDJM) | control | 0.125 | 0.052 |
|  | lim@chs | 0.567 |  |
| Fig. 6F | HNZZ control | 0.486 | 0.068 |
|  | HNZZ lim@chs | 0.087 |  |
|  | SDJM control | 0.101 |  |
|  | SDJM lim@chs | 0.122 |  |
|  | YNLC control | 0.280 |  |
|  | YNLC lim@chs | 0.225 |  |
| Fig. 6G | HNZZ control | 0.461 | 0.056 |
|  | HNZZ lim@chs | 0.318 |  |
|  | SDJM control | 0.827 |  |
|  | SDJM lim@chs | 0.526 |  |
|  | YNLC control | 0.067 |  |
|  | YNLC lim@chs | 0.472 |  |
| Fig. 6H | HNZZ control | 0.789 | 0.155 |
|  | HNZZ lim@chs | 0.115 |  |
|  | SDJM control | 0.244 |  |
|  | SDJM lim@chs | 0.146 |  |
|  | YNLC control | 0.302 |  |
|  | YNLC lim@chs | 0.204 |  |
| Fig. 6I | HNZZ control | 0.364 | 0.442 |
|  | HNZZ lim@chs | 0.928 |  |
|  | SDJM control | 0.976 |  |
|  | SDJM lim@chs | 0.711 |  |
|  | YNLC control | 0.707 |  |
|  | YNLC lim@chs | 0.790 |  |
| Fig. 6J | HNZZ control | 0.820 | 0.126 |
|  | HNZZ lim@chs | 0.670 |  |
|  | SDJM control | 0.559 |  |
|  | SDJM lim@chs | 0.524 |  |
|  | YNLC control | 0.451 |  |
|  | YNLC lim@chs | 0.524 |  |
| Fig. 7C | HNZZ control | 0.833 | 0.234 |
|  | HNZZ lim@chs | 0.265 |  |
|  | SDJM control | 0.230 |  |
|  | SDJM lim@chs | 0.265 |  |
|  | YNLC control | 0.126 |  |
|  | YNLC lim@chs | 0.506 |  |
| Fig. 7D | HNZZ control | 0.728 | 0.156 |
|  | HNZZ lim@chs | 0.171 |  |
|  | SDJM control | 0.915 |  |
|  | SDJM lim@chs | 0.613 |  |
|  | YNLC control | 0.512 |  |
|  | YNLC lim@chs | 0.989 |  |
| Fig. 7E | HNZZ control | 0.247 | 0.126 |
|  | HNZZ lim@chs | 0.089 |  |
|  | SDJM control | 0.769 |  |
|  | SDJM lim@chs | 0.758 |  |
|  | YNLC control | 0.583 |  |
|  | YNLC lim@chs | 0.376 |  |
| Fig. 7F | HNZZ control | 0.820 | 0.677 |
|  | HNZZ lim@chs | 0.670 |  |
|  | SDJM control | 0.559 |  |
|  | SDJM lim@chs | 0.524 |  |
|  | YNLC control | 0.451 |  |
|  | YNLC lim@chs | 0.524 |  |
| Fig. 7G | HNZZ control | 0.444 | 0.079 |
|  | HNZZ lim@chs | 0.926 |  |
|  | SDJM control | 0.101 |  |
|  | SDJM lim@chs | 0.332 |  |
|  | YNLC control | 0.154 |  |
|  | YNLC lim@chs | 0.948 |  |
| Fig. 8A | control | None (All measurements = 0) | 0.116 |
|  | IMI | 1.000 |  |
|  | [IMI@MSNs](mailto:IMI@MSNs) | 1.000 |  |
|  | [IMI@MSNs@lim@chs](mailto:IMI@MSNs@lim@chs) | 1.000 |  |
| Fig. 8B (0) | IMI | 0.115 | 0.615 |
|  | [IMI@MSNs](mailto:IMI@MSNs) | 0.254 |  |
|  | [IMI@MSNs@lim@chs](mailto:IMI@MSNs@lim@chs) | 0.295 |  |
| Fig. 8B (2) | IMI | 0.162 | 0.970 |
|  | [IMI@MSNs](mailto:IMI@MSNs) | 0.549 |  |
|  | [IMI@MSNs@lim@chs](mailto:IMI@MSNs@lim@chs) | 0.245 |  |
| Fig. 8B (4) | IMI | 0.226 | 0.654 |
|  | [IMI@MSNs](mailto:IMI@MSNs) | 0.713 |  |
|  | [IMI@MSNs@lim@chs](mailto:IMI@MSNs@lim@chs) | 0.202 |  |
| Fig. 8C (back) | IMI | 0.302 | 0.340 |
|  | [IMI@MSNs](mailto:IMI@MSNs) | 0.605 |  |
|  | [IMI@MSNs@lim@chs](mailto:IMI@MSNs@lim@chs) | 0.880 |  |
| Fig. 8C (frontal) | IMI | 0.345 | 0.060 |
|  | [IMI@MSNs](mailto:IMI@MSNs) | 0.586 |  |
|  | [IMI@MSNs@lim@chs](mailto:IMI@MSNs@lim@chs) | 0.191 |  |
| Fig. 9D (25) | control | 0.254 | 0.375 |
|  | IMI | 0.685 |  |
|  | [IMI@MSNs@lim@chs](mailto:IMI@MSNs@lim@chs) | 0.899 |  |
| Fig. 9D (50) | control | 0.777 | 0.101 |
|  | IMI | 0.280 |  |
|  | [IMI@MSNs@lim@chs](mailto:IMI@MSNs@lim@chs) | 0.856 |  |
| Fig. 9D (100) | control | 0.997 | 0.482 |
|  | IMI | 0.793 |  |
|  | [IMI@MSNs@lim@chs](mailto:IMI@MSNs@lim@chs) | 0.709 |  |
| Fig. 9D (200) | control | 0.981 | 0.550 |
|  | IMI | 0.779 |  |
|  | [IMI@MSNs@lim@chs](mailto:IMI@MSNs@lim@chs) | 0.900 |  |
| Fig. 9D (300) | control | 0.659 | 0.108 |
|  | IMI | 0.576 |  |
|  | [IMI@MSNs@lim@chs](mailto:IMI@MSNs@lim@chs) | 0.941 |  |
| Fig. 9G (25) | control | 0.254 | 0.086 |
|  | IMI | 0.304 |  |
|  | [IMI@MSNs@lim@chs](mailto:IMI@MSNs@lim@chs) | 0.124 |  |
| Fig. 9G (50) | control | 0.777 | 0.101 |
|  | IMI | 0.737 |  |
|  | [IMI@MSNs@lim@chs](mailto:IMI@MSNs@lim@chs) | 0.803 |  |
| Fig. 9G (100) | control | 0.997 | 0.304 |
|  | IMI | 0.839 |  |
|  | [IMI@MSNs@lim@chs](mailto:IMI@MSNs@lim@chs) | 0.961 |  |
| Fig. 9G (200) | control | 0.981 | 0.730 |
|  | IMI | 0.814 |  |
|  | [IMI@MSNs@lim@chs](mailto:IMI@MSNs@lim@chs) | 0.417 |  |
| Fig. 9G (300) | control | 0.659 | 0.168 |
|  | IMI | 0.856 |  |
|  | [IMI@MSNs@lim@chs](mailto:IMI@MSNs@lim@chs) | 0.376 |  |

^a^ The data were considered to have homogeneity of variance across groups when the *P*-value from Levene's test exceeded 0.05.

^b^ The data did not significantly deviate from a normal distribution when the *P*-value from Shapiro-Wilk test exceeded 0.05.

Table S4 FDR Correction of the Data.

| Analysis | Group | *P* Value | *q* Value (FDR corrected)^a^ |
| --- | --- | --- | --- |
| Fig. 4C | HNZZ | 0.001 | 0.003* |
|  | YNLC | 0.001 | 0.0015* |
|  | SDJM | 0.004 | 0.004* |
| Fig. 4E | HNZZ | 0.017 | 0.0255* |
|  | YNLC | 0.05 | 0.05* |
|  | SDJM | 0.001 | 0.003* |
| Fig. 4F | HNZZ | 0.006 | 0.009* |
|  | YNLC | 0.001 | 0.003* |
|  | SDJM | 0.226 | 0.226 |
| Fig. 4G | HNZZ | 0.001 | 0.003* |
|  | YNLC | 0.012 | 0.012* |
|  | SDJM | 0.001 | 0.0015* |
| Fig. 4H | HNZZ | 0.001 | 0.003* |
|  | YNLC | 0.005 | 0.0075* |
|  | SDJM | 0.025 | 0.025* |

^a^ For multiple comparisons, statistical significance was determined after False Discovery Rate (FDR) correction, with a *q*-value < 0.05 denoted by an asterisk (*).

Table S5 Toxicity of different pesticides to *Coccinella septempunctata*.

| Treatment | Slope ± SE ^a^ | LC_50_ (95% CL ^b^) (mg/L) | *P* | SR ^c^ |
| --- | --- | --- | --- | --- |
| IMI | 1.48 ± 0.31 | 12.91 (6.26 -19.20) | 0.99 | 1 |
| IMI@MSNs@chs@lim | 1.12 ± 0.21 | 46.50 (24.15-74.89) | 0.99 | 3.60 |

a Standard error.

b Confidence limits

c SR synergism ratio at LC_50_ value=LC_50_ value of IMI@MSNs@chs@lim to *C. septempunctata* */*LC_50_ value of IMI to *C. septempunctata.*

The reference sequence of genes.

>gene3895 rna5585

CGGAATGTTATCTTTGTTACATTAGATCGTATACAGCATTAGTGTCCACTTTAAAGGATTGCGGTGTTGACTTCTGTCGGTAAGTTGTTTGTCGTATTCCGTGCAAACGATCTCCGGCCATGTTCACCACCGATTGGTGGATAAATGTCGTTACGGCATGCACGATAATAGTGACGATCGTCTATTATTTCTGCGTGTCGACCTTTCAGAAATGGGAAAAGCTCAACGTGCCGTACATAAAACCGATCCCGTTGTTTGGGAACTTTTTGAACATAGCCTTGGGCAAGGACCATCCGCTGGAATTTTACAACAAAATCTATAACGAGTTCGAGGGTCGCAAATATGGAGGACTGTTCCAGATGAGAACGCCTTATTTAATGGTCCGCGATCCCGAAATAATCAACGACGTGATGATAAAAGACTTCTCGTCGTTCCCCGACCGCGGAATTTACTCGGATTTCGCGGTCAACCCGTTGTCGAACAACCTGTTTTTCATGGAAAATCCTCAATGGAAAACTATAAGAAACAAATTGACCCCCGCTTTCACGTCGGGAAAGCTCAAGACAATGTACGATCAGATCAAAGAGTGCGGAGACGTATTGATGAAAAACGTCGATATCAAATTAAATGAAAACAACAACGAAATAGAAATAAGGGACATCATGGGAAAGTATTCTACTGACGTCATCGGCACTTGCGTTTTTGGCCTCAAGTTGAACGCCATAACCGATGACGAATCCCTATTTCGTAAGTACGGCAAATCGATATTCACACCTTCAATGAGAATGCTTTTCAGAGAATTGTGTTTGATGATTACTCCTGCACTTTTGAAAGTCGTAAGAGTGAAAGATTTTCCAACGGATGCGACTGACTTTTTTCACGAAGCGTTCAAAGAAACGCTGACTTATAGACTTGAAAATAAAATAGTCAGAAATGACTTCGTTAACTGTTTAATGCAAGCAAGAAATGATTTAGTGTTGAATAAAGATTTACCTAAACATGAAAAATTTACTGAATCGCAAATCGTTGCAAATGCTTTCGCAATGTTTGCTGCTGGATTTGAAACTATATCCACTACTATAAGTTTTTGTTTATATGAATTAGCATTAAATAAATCTATACAAGACAGAGTACGCCAAGAGATTCAACTAAAACTGTCCAAAAATGACGGACAAATTAACCATGATTTTTTGATGGATCTTAATTACTTGGATATGGTTATAGCAGGTAATTTATTTATAGTTCATAATTGTTTATAATACCGACCAGGCCAGAATCTATCATCGTCAACCACCGACTGGTGGATTTATATCGCCTCGGCGTGTTTAGTCGGGGTGACGATCACCTATTACTTTTGCATTTCAACGTTCAGTAAATGGGAAAAACTCAACGTGCCCTACATCAGGCCGATTCCGTTGTTCGGAAACTTTGTGAGAGTAGCTTTGTCAAAAGACCACCCTTTGGAGTTTTACAACAAAATCTACTACAAGTTTGCTGGTCTAAAATACGGAGGACTGTTCCAGATGAGGACACCGTATTTGATGATTCGTGATCCAGAAATAATCAACAACGTGCTAATAAAAGACTTCTCGTCTTTCCCAGACCGTGGTATTTACTCGGATTTAGCGGCGAATCCATTGTCGGACAACTTGTTCTTCATGGAAAATCCCCGATGGAAAACAATAAGAAACAAATTGACCCCCGCTTTCACGTCGGGAAAGCTCAAGACGATGTACGATCAGATCAAAGAGTGTGGAGACGTATTGATGACAAACATCGACAAGTGTTTAAGGGGGGGAAACGAAGAAATAGAAGTAAGAGACATCATGGGGAAGTATTCGACCGACGTCATCGGCACTTGCGCTTTCGGGCTCAAGCTGAACTCCATAAGCGATGATGAATCCCCATTTCGAAAGTACGGAAAATCGATATTCATACCTTCACTAAGAACTCTTTTCAGGGAGCTGTGCCTGATGGTGACCCCCTCACTTTTGAAAGTTGTAAGGGTGAAAGATTTTCCAACGGATGCGACTGACTTCTTTCACTCGGCGTTTAAAGAAACGATAGCGTATAGACTTGAAAATAAAATAGTCAGAAATGACTTCGTTAATTGTTTAATGCAGGCAAGAAATGAATTGACTTTGAATGCAAATTTACCCAAAGAAGAAAAATTTTCCGAATCACAAATTGTAGCAAATGCTTTTGTAATGTTTGCTGCTGGGTTCGAAACAACATCAACTACTTTAAGTTACATCTTATATGAATTAGCGTTGAATACGTCTATTCAGGACAAAGTACGTCAAGAGTTTCAGTTGAAATTATCCAATAGTGATGGACAAATTGACAACGAATTTTTGATGAGTCTTAATTACATGGATATGGTTATTGCGGAAACCCTCCGTAAGTATCCTCCTTTAATTGCTTTATTCAGAAAAGCATCACAAACATACCGTTTACCTGACAACCTAATACTGGAAAAAGGCCAAAAAATAGTAATTCCAATTTACTCACTCCATTTCGATGATAAATATTTCGAGGATCCTCAAAAATTCGATCCTGAAAGATTTTCACCCGAAAACAAAGATAAACGTCCTAATGGTGTTTATCTTCCATTTGGTGATGGACCTAGAATGTGTATAGGAAAACGTTTTGCTGAGATGGAAATGAGATTGGCTTTACTCGAAATGTTGAGCAAATTTGAAGTCCTACCATGTGAAAAAACAGAAGTTCCTCTAAAATATTCTAACAAAGTTTTAACATTGATGCCAAAACATGGAATTTGGTTAAAATTTCAAAAAATTGCTTAACTTTAGTTAAGAATTATGTATTTGAACAAAAAAAAAATTAACAGTTCGTAACCTGAGAAAATTGTGTGGATTTTACTATATATGATATATATTATATTTGTATAATTTCAAAAATATTATAATAATATGTATTGAATTTGTAAGTATACAATCTTTATTTTAAATA

>gene4273 rna6114

ATGATTTCCGAGATGTGGTGTTCGACCATCGAACTGTTTAGCTCCGTGAACTTATTGTGCATGGCAGTCTTTCTATCGATCGTCTACTACTTAACGATGTCCACGTACGATAAATGGAGCAAACTGAACGTGCCCTACGTAAAGCCCGTGCCGCTGTTTGGAAACTCAATGAAAATGGTCTTGAAAATGGAGCACCCGTTGGACTTTTTCGGTCGTATTTACAACCAGTTCCCGGACGCGAAGCTTTGCGGTTTCTATCAAATGACCACCCCATTCCTGATGATTCGTGATCCGGAGCTGATCAACGCCATGATGGTTAAGGACTTCTCATACTTTACCGACCATGGTTTTGATACAGACCCATCTGTCAACCTCATGGCCAGCAGCCTTTTCATGTTGAATGGCGATCGGTGGAGAACAATGCGACAGAAGCTCAGTCCCGGATTCACATCTGGAAAACTGAAGGACACTCATGACCAAATCAAGGTCTGCATCGACCAGCTAATGAACGTTTTCGAAGAAAACCTGAAAGTCAGCGATCACTTCGAACTACGAGAGTTGATTGGAAACTTCTCGACGGATGTCATCGGTATGTCCGCTTTCGGTCTGAAACTAGACACGATCAAAAACGGCAATACGGACTTCCGCATGTTTGGCAAGAAAATATTCCAGGCGGACTACAAACAGCTCTTTGTCCAGGCTATGCTGCTGTTTTCGCCAAAGCTGGCTTTAGCTCTTAAGCTGAAACAGTTTCCGGAGGACGCCGCTAACTTTTATGAATCTATGTTCAGGGACGTCCTCGAGTATAGGGACAAGAACAACGTCGTCAGAAACGACGTCACTCAAACCCTGATTCAAGCTAGAAAAGATTTGGTGAAAAATAACGACGGTGACGAACCGACATCCGAAGATAAATGGACCGAAATGGACATAATCGGAAACGCGGTACTGATGTTTGTCGCTGGTGCCGAAACTGTTTCCATTACAATATGCTTTTGTTTGTACCAGTTGGCATTAAACAAAGATATCCAAGATAGACTGCGTGAAGAGATCGTTATGGCAAAAGCGAAAAACGGTGGAGAGTTGAACAACGACTTTTTGATCAATCTCCACTACATGAATATGGTTTTAGAAGAAATTTCGCGCAAGTATGCCATCACCATGATCATATTCAGACGAGCGACGAAGAATTACCAAGTACCCGGTACCTCATTAGTCATTGAAAAGGGACAAAAGATCACCATACCAGTGTACTCCATACATAATGACCCGAAATATTACCCCGATCCCGATACTTTCGATCCTGAAAGATTTTCGACGGAAGAAAAAGCCAAACGGCGTAATGGCACTTACTTGCCGTTTGGCGATGGGCCGAGATTGTGCATAGGTAAACGGTTGGCGGAATTAGAAATGAAATTGGTTCTATCAAAAATATTGTTGAAATATGAAGTCTTTCCGTGTGAAAAAACAGAAATTCCACTCAACATACGAGGTCCTGGAAGTATCGTCAGTCCGAAAAATGGTATTTGGTTGAGCTTTAAGCCGATTGCCACAAATTAACATATATCAATAATATGCATATTGAATGTCTGTTTTTTTCAATATTACAACATTTATTTAAAATACAAATCACAATATTTTTTAATACTCAAAACTCATGTATAATTACACGTTTCTATTATAAGAACAATAACATTGAGTAGTAACTATGAATATAATATATATATCATATATGTCATGCCAGTTATTGGTCAGACTGTTCCTTTAACTTTAGGAACAGAATTGTAATCGAGATTTTCTAAACATATATTTCTATAATATGTTATAATACACTGAAATATTAATACGTACAAATAAATAAAATACTAAATATAGGTAATCGATGATACTATATTCTGTACTCATAAGTTTCATATTACCGATACTGACATATTTTGTTGTTTAATTAATTAATACGATTAATAGAAAACTGTAAATTAGTGATTTACTTATGTTTGATGTACCTACTATGTGGTATAAAAATGTCTATGGTTATTTTATACGTGGGTCTTAATTTACTTTATAATATTATATGAAAAATAGTGATATTTAGATTTTAA

>gene5869 rna8526

GCTGGGTGCAATTCTGGTCAGTCGTGACTTGTGGGTGATAAATGCGAATTTTGCAGATTCGGGAACAATACTATAATATTTACAATCTTCGAATACTATGATTTTATGGATGATTAATTGTCTGTCTGATTCGTTTACTCTGATATGTACCACAGTGGTGGGTTTACTGTTCTACTATTATTCGACAGCCACGTACGAAAAATGGAGGAAAGCTAACGTGCCGCATTTGAAGCCGGTACCGTTGTTCGGGAACTTTTATAGAACGACGATGATGCTTGAAAGTCTTAACAATACGTACGACAAAATTTACAAACAGTTTCCAGATGAAAAAATGTGTGGGTTTTATCAGATGAGGACGCCTTTCTTGATGATTCGAGATCCGGAAATTATAAACAATGTGCTCATCAAGGACTTTTCGCACTTCACAGACCGTGGGTTTGAAATGGACCCATCTGCTAACTTTTTGGGTAGCAGTTTGTTCTTCACAAATGGCCAAAAGTGGAAGATCATGAGACAAAAAATAAGCCCGGGATTTACATCCGGTAAACTCAAGCTGATGCACAGTCAGATTAAAGAATGCAGCAAAGAGATGATAAATTATATCGATAGGAAATCGAAGACGACTGATCAGTTTGATGTGCACGATATCATGAATAAATATGCCACTGACGTAACTGGGACATGTGCTTTCGGTTTGAAATTAGGCAGTATGACAGACGAAGACAACGAATTTCGAAAGTTTGCGAAATTGATATTTAAACCATCGTTTAGGCTAATTTTTGCCAACACGTTGGTAATGATTTCACCAAAAATATCGAGAATATTAAAAATAAATCCCACCCCACCAGAAGTTGAGGATTATTTTATTTCATCATTCAGAGGTGTAATCGAGTACAGAGAAAAGAACAATGTGAATAGGAACGATTTGGCCCAAACATTAATGCATGCTAGGAAAGAACTGATACTAAATAATAACTCGTATCCTGAAGAGAAATTTACAGAAATGGATATTATTTCAAATGCAATTATAATGTACTTTGCTGGTGCCGAACCGGTATCCGATACGTTGGGATTTTGTTTACATGAGTTGGCAATAAATAAACAAGTACAAGACAAATTGCGTGAACATATCATCACGAAGATGGAAAAACATGGTGGTGAATTTACCAACGATTATTTGATGGACCTCCATTACGCTGACATGGTTTTATCAGAAACATTGCGCAAGTACAACGGGTCACTTAATATATTTAGAGTAGCTACTCAGACATACCAAGTCCCGGACTCGTCATTAATTATCGAAAAAGGTCAACAAATATTAATACCGGCTTACAGCATTCATCGTGATCCAAAATATTATACTAATCCTGATGTTTTTGATCCGGAAAGGTATTCTCCGGAAGAAAAATTAAAACGACCTAGTGGCACTGATTTGTTGTTTGGGGACGGGCCTCGTTTTTGTATAGGTAAGCGTTTGGCTGAATTGGAAATGAAATTAGGTTTATCAGAAATAATTTCGAAGTTCGAAGTTTTGCCGTGTGAAAAGACTGAAAATCCCATTCAATTGGCAGCTGGAGATGCAATTAGACCGAAGAATGGAATTTGGTTGAGTCTGAAACCAATTGTTGTTAATTGTTAATCATACCTGGATATATATAATAGAGTAAAATATAATTGGGTAGAGTCCATATAAAAAACATGCATATTAAAAATTAAAAAATTATATTCAA

>gene7532 rna11121

AAAACACACACATAATTGTGTCAAATCTATATTGTTTATGATGTCATTGCTCAAAAAACCGCATGAGCCCATCGCATGACCTTGTTCACCTGCACCAGCAAAACGAAACCAATAATCACGGAAGAGATGGAAAACGTAAATTAGGCTCGCTGTATCAACAAGCGTAGTATATGCGTAACACAACGCAGTACTCGTTTGAACTTTCACGTGGGCCGCAGCTGTAGTAATTTTTCCTAAAAGTTTTTGAGCCAATAACTGCAGCTGTCGTAACAAGTCATTCATGATGTTATTTCTGCCTGACTGGTTCCTCGACAACTTCACATTTCTGAGTTTGATCGCCGTTTTCGTGAGCTTCTACTATTATTCGACATCGACGTACGGTAAATGGCAGAAATTGAACATTCCGTACTTACCACCAGTGCCACTGTTTGGCAACACGTTCAGAATGATGTTGAACCTCGAACATCCAATTGACACGTTTGAAAGGTTCTACTACAGTTTTCCAGACGCAAAAGTCTTCGGATTCTACCAGATGAGGGAGCCGATGCTGTTAGTCCGTGACCCGGAGCTCATCAACAGGATACTGGTAAACGATTTTTCATACTTCACCGATCATGGTATGGACATGGACCCGTCCTCGACTGTGGTAGCCAACAGTCTGTTTTTCACCAATGGCAAAAAATGGAGGACAATGCGCCAGCAACTGAGTCCAGGCTTTACGTCCGGCAAGTTAAAAGATGCGTATTTTCAAATCAACGAATGCGGCAACGAGATGGTATCTGGCCTTGTCGAGAAGCTAGGGAAAACTAACATTATAGAAGTGAAAACGATGACTGATGGTTTTTCCACAGACGTAATTGGAACTTGCGCGTTTGGTCTGAAACTGGATGCGATCAAGAATGATGAGTCGGACTTCCGCCGGTACATTAGATTGTTTTTCCACAGCTCCTGGAAACAGATGATTTTCCGGGTGATGGCAATGACCAGCCCCTGGGTGAACAAGCTATTAAAATTACAAATGTTTTCTGAAGAAGCTACCAACTTTTTCTATAAGGTGTTCACTGACGTCGTCAATTACCGTGAAAAGCACAATGTGGTCCGGAACGATTTGGCGCAAACATTGATGCAAGCGCGAAAAGAATTAGTGTTGAACCCGACCTCCGAAG

>gene8018 rna11857

GTATATAAGGGCAAGTCCGTCTCGGTCCAAGTATCATTCGCTCATCAACAGTCAGTAAAGCAATATCGACTTCTTTCAAATCATCAACCAACCGAACCAAACAAATATCAAAAATGAACGCCATCATGAAAATCACATTAGCCGCCGTCGCAGTGTTCGCGTCCACCATGATGACCGCTTCGGCCAAACCATCTGGACTCGTGCCCGCCGCCGCAATCGCACCGGTGGCCGTCGCCGCGCCTTACGCTAGCTCGTTCACGTCACACAGCGTGGCCCACAGCGTGGCCACTCCGGTGTTGCCGGCCGCTCCGTACGTGGCAGCCGCCCCGTACGCCGCGGCTCCCTACGTCGCAGCCCCTTACGCGGCCGCCCCTTACGTCGCCGCCCCTTACGCAGCAGCGCCATACGCAGCCTCCCCTTACGGTTACTACCCCTACGCCGCTCCGGCTTACCTGTAAAGGCCGTGACGGACGATGACCACCACCACCAACACCACCACAAATCAGTCTGACGGACGAGAGACGACCGCAACGACGAAATCACCAACTGCATCATAAGCCACTGATTCTTTTCATATTTTATAAAATTCATAAAACATAAAACTACACGTCATGTCACCGCGATACAAACACAATGTGTATCGCGCGTGCACAACACAAAATGTATTATACTATATATAATATATATATAATGAAACACTATGAAAATTTTTTATAA

>gene9605 rna14384

ATAGTATACGCGAGTCGAAAATGCGTAAAGGAGCAACGAACACGTGTGTTTTGATTTTTATCTTTTAAACGATATTCCGTTTCGGTATAATATAATATTATAATAAACTATAATAATAATAGTATTAATAATTATATGTGCTGTATATGACGTTTGCGAGTTCACTAGTTATTATCATTACCTACGACGACCCGCGAGGATATCATCGGACCAACTAATTGGCATTTGTGAAACCACTTTCTTCGGTGGAAAAGGAAAGGATTCGATAGGGACTCGAGAACAATTTTGAAATGTTCTGCTGTAGTGCTGTCTGTTCCGTGTTATAGCGAACTGTAGCAAGCCGATCGAATTTCAACGGTCAACTTCCTTTTGAATTTCACGCCTCCTCGTCAAAATGATCTTCTCCAACGTGATTGGAGCCCTGACGTCGGATTCGAACACGCTGTGGATGGCGTTGCTATCACTCGTGGTCCTCGGAGTGTACTTTTTATTTTCCGACAAATTTTCAGAGAACCGTGGAAGACAAATTTCACTGTTGCCGTCGATCACGAAAAGCCAATGGGCCTCGTTGCTTGTGTCGCTAAAACTCGCCAGTTTTGGGCCAAGAGATATTTTGCCGTTTTTCGACAACGTTATCAAAAAATATGGGTCGCTGATTCACTTTAAAATCATAGCACGTCATTATATTATCATAAACGATCCGGACGATATAAAGGTACTGCTGTCGAGCGTACAACATATTACGAAAGGTCCTGATTACGAGATGCTTGAACCGTGGTTGAACAAAGGGTTACTGACCAGCACTGATAATAAATGGCATTCTAGAAGAAAGCTGTTGACCAACACTTTTCATTTTAAAATATTGGAAACTTACATGCCGTCGCTTAATAAGCACTCCCGTTCATTGGTCAAAAATCTAATCAACGCGTCAGACAACGGAAAATCCATAGCGGATATCGACTCCCATGTAACTCTTTGTGCTTTAGACATAGTGTGCGAAACAATTATGGGTGTAAATTTGAGGAGTCAAGAGGGTAAATCGATGGATTATGTTAAAGCAATCAAAAATGTCAGTCAGATACTGATCAAGCGTATTTTTACGTTTTGGTATTGGAACGAAATAGTTTTCAACCTCAGTAGCCTCGGCAGAGATTTTCGTAAATCCTTAAAGTTACTACACGATTTCACCGAAAATGTCATACGGGAGAGACGGAAAATATTAGAAAACGTAGAACAAAATAAACCCGATGAAAACGGAAAGAAACGGATATATTCGTTTTTGGACCTTCTTGTCGGTGTTTCCGAGGAAAACCCTGGTGCAATGACTGATAAAGATATACGAGAAGAAGTGGACACGTTTCTTTTCGAAGGCCATGACACGTCTTCAATAGCCATTACAATGGCTATAATTCATTTGGGGCTCGATCAAAACATGCAAAATTTGGTCAGAGACGAGCTTTGCGAAATATTCGGCGACAGCGACCGGGACGCAACAATGGAAGACCTCAAAGCGATGACAAATTTAGAAAGAGTGATAAAAGAGACAATGAGACTTTATCCGAGTGTGACGGGTATCACGAGAACCCTCAAAGAGCCGCTCCGCCTCGACAAGTATACGATACCTTCTAAATCGGTGATGGTCGTCGTCCCTCATCTATTGCACCGCGACGAAAACATCTATCCCAATCCGGAACAGTTCGATCCCGACCGATTTTTGCCCGAACAGTGCAACGGACGCCATCCTTACGCATACATACCGTTCAGCGCCGGCCCCAGAAATTGTATAGGCCAGAAGTTTGCCATGTACCAGATGAAAACAGTATTATCCACGATTTTGAGGTACACGATTGTAGAGACGTTGGGGACGCAACAGAGCATCGTCATCAGCACACAGTTGATAATGAGAGCGGATTACTTGCCCAGTGTAAAAATAACTCCAATCTCCAACACAAATTTCGCAAGTCATATATTGTAATATATGGCATACATATAATAAATAAGTTAACTATTAATAATATCATCATAATAATTTAATCTAAGTGTAAATATCGTGTAATTACATAACTATACATATTTTGCATATAACGCAATACATTTTTTGTTTTTTATAATTATAATAATCTGCATGGGTGCAACTATGCATGGGTTTGGTTCAGACCAAATATTATATATATTTTATGTATCATATATTATGTTGGATAATAATAAATTATCGTCACAAAAAGTA

>gene13929 rna21338

ATGGAAGGGATGCTGCGACGAATTTTTAGCATAAATACCACAGCTCTCGTTTCTATAACCACTAGTTCATTTCTGTACACAGTCATAGTATAATTTGACCAAAAAAACCAATACAACACTAAAATGGCCGCTAAGTTCATCATCTTCGCCGCTTGCGTGGCTACCGCCCTTGCCCAATACTCCGCTCCAGCTTACAAGCCAGCGTACTCTGCGCCCGCTTACTCAGCACCAAAGGCATACGCCCCAGAACCTGCATACGCACCCACACCGTACAACTTCGAATACAGCGTAAACGATCCACACACCTACGATGTGCACAGCCAATCCGAATACAGTGACGGAAACGGTTACGTCAAGGGATCCTACAGCCTCGTCGAAGCCGACGGCTCCATCCGCACCGTCGAATACACCGCTGACGACTACAACGGTTTCAACGCCGTCGTCAAGAACGAAGGTGGATACAAGGCCCCGTCATACTCTGCCCCATCATACTCCGCACCAGCCTACAAACCAGCATACTCTGCACCTGCATACTCTGCCCCAGCCTACAAGCCAGCATACTCTGCACCAGCATACTCTGCACCAGCTTACTCTGCACCAGCTTACTCTGCACCGGCCTACAAGCCAGCATACAAGCCAGCATACTAATTTTTTAATTAGATGTGTAAACAACTACTCTTCATCGCCTTTTTTTCATGACTTCTTCGTGTACACCTCTGGGCCATCGTGTTCATATTGAACCATGGTTTTAAAGTCATAACATGACATTCCAATTTGATAATTGGTACAACTTACGTATTTATTGTGTAAATATATATAACAATGTGTATTTATTGAA

>gene13931 rna21340

TCTTCGATTTAAATACTGCGACAACAACCACGAAATTTATCAGTCATCGAGTTGTATTTGTCGCCGACAGAACAAACACAATATTTTCTCACCGTTCAAAACGACATCCATTCAAAATGATCGCCAAGGTATTCTTCATTGTCGCTTGCGCGTCCATCGCCGCCGCCCAGTACGCAGCTTACGCCCCGGAGCCCAAGTACGCACCCATCCCATACAGCTTCGAGTACAGCGTCAACGACCCACACACCTACGACGTCAAGAGCCAACGCGAATCGAGCGACGGAAACGGCAACGTCAAAGGCTACTACAGCCTTTTGGAAGCCGACGGTTCCACCCGCACCGTCGAATACACCGCTGACGACTACAACGGATTCAACGCCGTCGTCAAGAACTCCGCCCCAGCTGCCTACAAGCCAGCTTACTCCGCACCCTCGTACTCTGCCTACAAACCGTCATACTAATAGTTTTTTTTTTTTTCCCCAAAACATTCTAATATGTGATCCCCAACAACGTCTATCGCTTCTTCGTCTCATCATGTTTTCATCACGCGCACGCCTTCCGAGGACATAATATTATTATTTGTGAATCGCTGTCGCAATAATACTATACCGATGTAGGTACACGACAACGGGTACGATCTGCTGTGGGCGGGTGTGTTTTATTGTCACCGCCGCCGTCGAGACAATTTAAGACGGACACAGAGCACCGAAACGGAGCTCTGGCACAA

>gene13936 rna21344

GCGGACGAGAACCTGTGGCATATAAATACGGCGATCGCCATGGACAGCTTCACCAGTCAACGATCTCGTCGTCAAGAGTTGTATCCCGAAGAAACGAAAAACTCAAACACACACAAACCAAAATGGCAGCTAAGTTGATCATCTTCGCCGCATGCGTGGTTTCCGCCATCGCTCAATACCCCGCCCCGGCCTACAAGCCCGCTTACCCAGCCGCTTACCCAGCACCCGCTTACTCGGCACCAAAGGCATACGCCCCAGAGCCCGCTTACGCCCCGGCCCCATACAACTTCGAATACAGCGTCAACGACCCATCCACATACGACGTCAAGAGCCAGTCCGAGTACAGCGACGGAAACGGCAACGTCAAGGGATCGTACAGCCTGGTCGAAGCCGACGGTTCCACCCGCGTCGTCGAATACACCGCCGATGACTACAACGGTTTCAACGCCGAGGTCAAGAAAATCGAAGGAGGATACAAGGCCCCATACGCCGCCCCAGCCCCAGCCTACAAAGCCGCCCCGGCCTACAAGCCCGCTTACGCCGCACCAGCTTACCCAGCACCCGCCGCCTACCCAGCACCTTCATACTCCGCACCCGCCTACAAGCCAGCCCCGTACAAGGCTTACTAATAACCGACTCCTCGTCAGCACTCCCAGGCCACCTGTCAGTCATCGTCGCCGGTGAAACCAACACAATGTGACCGTTACCTACACCATACCGCGAGTCACATATCGTGTGTGTACAACTCGCGTATAAATATCATATATATTTTTTGTAAATAATTTATTACACGACGTACTATTATATTATTATATTGACCATCGTAATACTATATAATAA

>gene15901 rna24433

AATATAAAGTTTTTACCATTTAATTTAAATTATAATAACAATTATATGGAATACAAATTATACACTGTTAATTGTGTTAATACGTGTATACGACGTATACAATTATTTTTGGTGTACCTACCACATGAACGTAACACTAAGAACTTTTCTAGTTTCTAGTTGTATGAGAGCAGTGACAGTCGACAGATCCGAGGAAAGTCAACTGATATTGTCATATTGATGTTTTTCTTAAGATTAATGTTAGTAATATAATTAATATAATAATATGACAAATGTCATATTAGTATGATATTTTATCATTATTCCCATACTGAATATGTGGACGCATGGGTACTTTTTTTAATAATATATTTTATTTTTTCAGTGATCGTATTTCAAATATTGAAATATTAATATTATACTATATAACATAATACTATATAATGTCTCACCAAGGATTTGTGATTGATTTTAATAAGTTATATCTAACTTATAGCCTAGAATTTTATTCTACACTTTTATCACCAATTTATTACTATATAACGATTAATACAATATAATAAATTAAAGATTACCTAATTATATCTTACGAGTTGGTACCTATACTAGTTTTTATATTACTCGTAGACTAATTTATTTCGAAGGTATGCGATGTTTTAATATTCGACTCAATAAAATATTATTTTACACGGGTCGAATAAAAATATTATCAAGTTGGAGGTCAGTCTTGTATTAACAATTTAAAATACAACTGCATGCAGGGGACTCACGGGTAAAAATTAATGAGTTTTTAAGTATGGGCGATGTAGACGAAATTATCTTTGTTTTTCGATCTGCCGATGTAGACGTCATTTGACCTAATGTGTCTACATATTTCATTTCGAAAAATAAGGTATTTTTGTTATCGAAAAAAACTTATAAAATCGATATGTACATCGTTCACACAGTCAGTTCAGTTACGGGAACCATTACTATTGGACATTCTTATAAGCAACCTTAATTTTTTTTCATCAATCGACGAAAAACAAAATGAATACAATCACGATAGTAGTTTTGGCGGTGTTCGTCTTCTCGCAATGGTCGACCACTGCTGCGGTGACCACATATTCGACAGAAAAACCAAAGACCCCAGCCGTAGCGCAACTTTTGAGAAACGACTATGTCTACGATAACAGTGGACAGTTTAGCCTCAATTATCAGGTGGATGATGGAACATCGCAGACCAGGGAAGGTACGTTGGTTTTGAACGACGAAGGAGACGATTACGTTTTGATTCAGAAGGGTTCATATTCATACATTTCTCCCGAAGGCATCAAAGTTACGGTGACATACACAGCCGACAAGGAAGGTTTCAAGATTGTTGAATCTAGCAACGACGTCCCCGCCAGAGTGTAACTGGAAAGCCCATAGTCCATAGTCATATTATTATGTCCATAGCATCATTATAATATTGTATAAGGTTTAGTGCTTTGTTCATTATTATCTTAAACTGATTATGTAGTGTTCGCAAATAGATAGTCGTAATGTAGAATCAATCACGTATATATTTGAGTGTACATGTAATAAAATATACAGGGTATTTACCTATTAAAACATTTGAAACACATA

>gene16829 rna25749

ACAACTGTTCTTTGAAATAGATCACAAATCAATCAGCTTCTACAGCAAGCACAATACAATATTATCACTCATCTACATTTTGCAGACCAACTAAAACCAACCACCATGAAATCCTACGTAGCTGCCGTTTTATTGTTGGCTCTTGCCGTCTGCGCAACCGCCGAAGAAGCCAAGCAACAACAAGCTTCTCCTGCTAAGACAACCGACAAGCGCAGCATCTACGGACTGGGTTACGGCTACCCTGCATACCACGCACCCCTTTCCTACCCAGCTGCCCCCTTGGCTCTGCCCACATCCTTCATCTCGCCACTGAAGTACCACGCACCTCTGTCCTACCCTCTGTCCTACCACGCTCCCCTCTCTTACCACGCTCCCCTCCCCTATCACGCTCCCCTTTCCTACCACGCACCCCTCTCCTATCATGCACCCCTCCCCTATCACGCTCCCATCTCTTACGCATCCCCCCTGTCCTACCACCATGCACCAATCTACAAGGCCCCGTACTACGCCCCGTCTATCTACTAATAGATGATATCGCACTCGTCAACCGACACGCTATTGCAATAATAGGAAAAAATCATATAATCTGAACTATAATTGCTACGTCGTCGTCGTCGTCGTCGTCAATCTTTAATTCATGTATTTGTAAATCATTGTTAATAATCATATTATTTTATTTTATTTGTATATAATTATTTGTGCATCATCAT
